# Identification of differentially methylated genes as diagnostic and prognostic biomarkers of breast cancer

**DOI:** 10.1186/s12957-021-02124-6

**Published:** 2021-01-26

**Authors:** Xiao-hong Mao, Qiang Ye, Guo-bing Zhang, Jin-ying Jiang, Hong-ying Zhao, Yan-fei Shao, Zi-qi Ye, Zi-xue Xuan, Ping Huang

**Affiliations:** 1 Department of Pharmacy, Zhejiang Provincial People’s Hospital, People’s Hospital of Hangzhou Medical College, Hangzhou, China; 2 Department of Pharmacy, The First Affiliated Hospital, College of Medicine, Zhejiang University, Hangzhou, China

**Keywords:** Breast cancer, Methylation, Biomarkers, Diagnosis, Prognosis

## Abstract

**Background:**

Aberrant DNA methylation is significantly associated with breast cancer.

**Methods:**

In this study, we aimed to determine novel methylation biomarkers using a bioinformatics analysis approach that could have clinical value for breast cancer diagnosis and prognosis. Firstly, differentially methylated DNA patterns were detected in breast cancer samples by comparing publicly available datasets (GSE72245 and GSE88883). Methylation levels in 7 selected methylation biomarkers were also estimated using the online tool UALCAN. Next, we evaluated the diagnostic value of these selected biomarkers in two independent cohorts, as well as in two mixed cohorts, through ROC curve analysis. Finally, prognostic value of the selected methylation biomarkers was evaluated breast cancer by the Kaplan-Meier plot analysis.

**Results:**

In this study, a total of 23 significant differentially methylated sites, corresponding to 9 different genes, were identified in breast cancer datasets. Among the 9 identified genes, *ADCY4*, *CPXM1*, *DNM3*, *GNG4*, *MAST1*, *mir129-2*, *PRDM14*, and *ZNF177* were hypermethylated. Importantly, individual value of each selected methylation gene was greater than 0.9, whereas predictive value for all genes combined was 0.9998. We also found the AUC for the combined signature of 7 genes (*ADCY4*, *CPXM1*, *DNM3*, *GNG4*, *MAST1*, *PRDM14*, *ZNF177*) was 0.9998 [95% CI 0.9994–1], and the AUC for the combined signature of 3 genes (*MAST1*, *PRDM14*, and *ZNF177*) was 0.9991 [95% CI 0.9976–1]. Results from additional validation analyses showed that *MAST1*, *PRDM14*, and *ZNF177* had high sensitivity, specificity, and accuracy for breast cancer diagnosis. Lastly, patient survival analysis revealed that high expression of *ADCY4*, *CPXM1*, *DNM3*, *PRDM14*, *PRKCB*, and *ZNF177* were significantly associated with better overall survival.

**Conclusions:**

Methylation pattern of *MAST1*, *PRDM14*, and *ZNF177* may represent new diagnostic biomarkers for breast cancer, while methylation of *ADCY4*, *CPXM1*, *DNM3*, *PRDM14*, *PRKCB*, and *ZNF177* may hold prognostic potential for breast cancer.

**Supplementary Information:**

The online version contains supplementary material available at 10.1186/s12957-021-02124-6.

## Background

Breast cancer is the most commonly diagnosed cancer and the leading cause of cancer-associated death among women worldwide [1]. Early diagnosis and accurate prognostic assessment of breast cancer are crucial for timely targeted treatment [2]. Accumulating evidence suggests that DNA methylation may hold an important role for the development and progression of breast cancer [3–5].

DNA methylation consists in the addition of a methyl group to carbon 5-position of cytosine within a cytosine guanine (CpG) dinucleotide [6]. This molecular process is critical for several important cellular mechanisms, including embryonic development, regulation of gene expression, X-chromosome inactivation, and genomic imprinting and stability [7]. Aberrant hypo- and hypermethylation patterns of the DNA have been identified as critical players in tumorigenesis, promoting the expression or silencing of oncogenes and tumor suppressor genes, respectively [8–10]. Therefore, abnormal DNA methylation, acting as a cancer-related biomarker, could be helpful for cancer early detection and prognosis, as well as for predicting response to treatment of cancer.

DNA methylation markers are not currently in use in clinical settings for breast cancer assessment. This is mostly due to lack of evidence on their clinical value in large cohorts of breast cancer patients [4, 11–13]. Indeed, available data on the clinical potential of cancer-specific methylated markers rely on platforms with low genomic coverage, small sample datasets, or missing appropriate healthy counterparts for comparison [14].

In the present study, we aimed to evaluate methylation changes specific to breast cancer that could be used as tools in the clinical setting for diagnostic and prognostic assessment of patients. To achieve this goal, we used different bioinformatics approaches to analyze several publicly available methylation datasets of samples collected from cancer patients and healthy counterparts.

## Methods

### Description of breast cancer and control samples

**Fig. 1:**
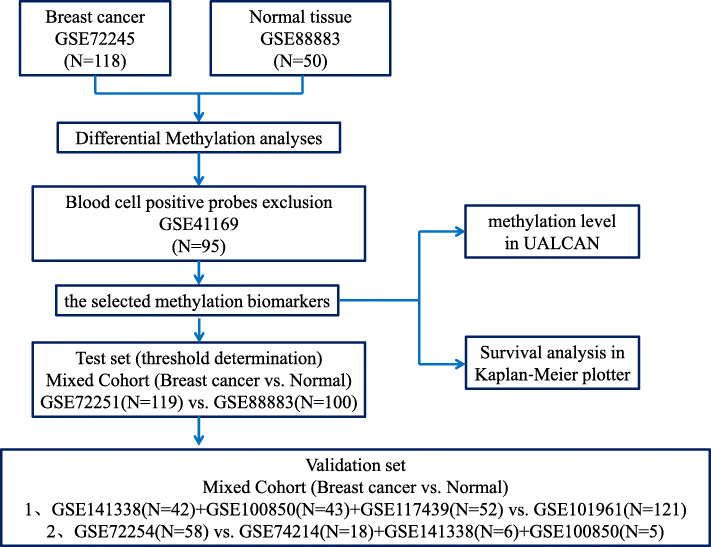
Workflow of the study. A multistep marker discovery analysis was performed to identify differentially methylated gene-based biomarkers of breast cancer

Breast cancer and control samples publicly available at the Gene Expression Omnibus database (GEO, https://www.ncbi.nlm.nih.gov/geo/) were used for the different bioinformatics analyses. Cancer samples were obtained from GSE72308, which includes three sets (GSE72245, *N* = 118; GSE72251, *N* = 119; GSE72254, *N* = 58), as well as from GSE141338 (*N* = 42), GSE100850 (*N* = 34), and GSE117439 (*N* = 52). DNA methylation data from normal tissue samples was used as control and was obtained from GSE88883 (*N* = 100), GSE74214 (*N* = 18), GSE141338 (*N* = 6), GSE100850 (*N* = 5), and GSE101961 (*N* = 121) datasets. Data from GSE41169 (*N* = 95) were used as a blood control dataset. Information of all samples is compiled and available in supplementary information (Fig. 1; Table S1).

### Differentially methylated analyses

Data from 118 breast cancer samples (GSE72245) and 50 normal samples (GSE88883) was analyzed by R package ChAMP, according to a previously described protocol [15]. Probe signal was removed when detected *p* value was above 0.05, or when more than 1% of the dataset contained no information. Briefly, differential methylation analysis was performed at probe (lmFit from limma; adjusted *p* ≤ 1 × 10^−35^; minimum delta beta value of 0.35) or region level (bumphunter from minfi; regions represented by at least two probes with *L* ≥ 2). In order to minimize the risk of false positive detection in blood tests, methylation in leukocytes was excluded (GSE41169; maximum beta value allowed = 0.1). Differentially methylated probes were limited to those overlapping differentially methylated regions, which was distant of a maximum 150 bp, not located in centromeres or telomeres. Lastly, methylation level of differentially methylated genes in control and breast cancer samples was plotted with the use of GraphPad Prism software.

### UALCAN database analysis

UALCAN online tool (http://ualcan.path.uab.edu) is designed to provide easy access to publicly available cancer transcriptome data (TCGA and MET500 transcriptome sequencing), including 793 breast cancer samples and 97 normal samples. Therefore, it was used to perform a comprehensive analysis of promoter DNA methylation patterns in control and breast cancer samples [16]. In this study, the beta value indicated level of DNA methylation ranging from 0 (unmethylated) to 1 (fully methylated), and different beta value cutoff was considered as hypomethylation [beta value 0.3–0.25] or hypermethylation [beta value 0.7–0.5]. Additionally, mRNA expression of the identified genes in breast cancer was also analyzed using UALCAN.

### Marker discovery analysis

Receiver operating characteristic (ROC) analyses were performed in GSE72251 and GSE88883 with the pROC package in R Bioconductor to establish thresholds, considering normal and adjacent mucosa as positive outcome and cancer as negative; only loci showing a threshold below 0.35 were kept. ROC curve was generated, and area under the curve (AUC) with the binomial exact confidence interval was calculated. For AUC values above 0.9, the differentially methylated gene was deemed able to distinguish between control and breast cancer with excellent specificity and sensitivity. AUC for the combined epigenetic signature was assessed using a logistic regression model [17]. Each threshold was used to stratify the two mixed cohorts, defining a positive predictive value and negative predictive value for discriminating normal adjacent from tumor tissue. The two mixed cohorts were as follows: mixed cohort 1 included breast cancer (GSE141338, GSE100850, and GSE117439) and control (GSE101961) samples, whereas mixed cohort 2 included breast cancer (GSE72254) and control (GSE74214, GSE141338, and GSE100850) samples.

### Survival analysis

Prognostic value of the selected DNA methylation-driven genes was evaluated through the Kaplan-Meier plot assessment (http://kmplot.com/analysis/) with data from the mRNA breast cancer database [18]. Median value of all gene expression levels was used as threshold to identify and separate cases with high or low gene expression. *p* < 0.05 was considered significant.

## Results

### Identification of differentially methylated genes

**Fig. 2:**
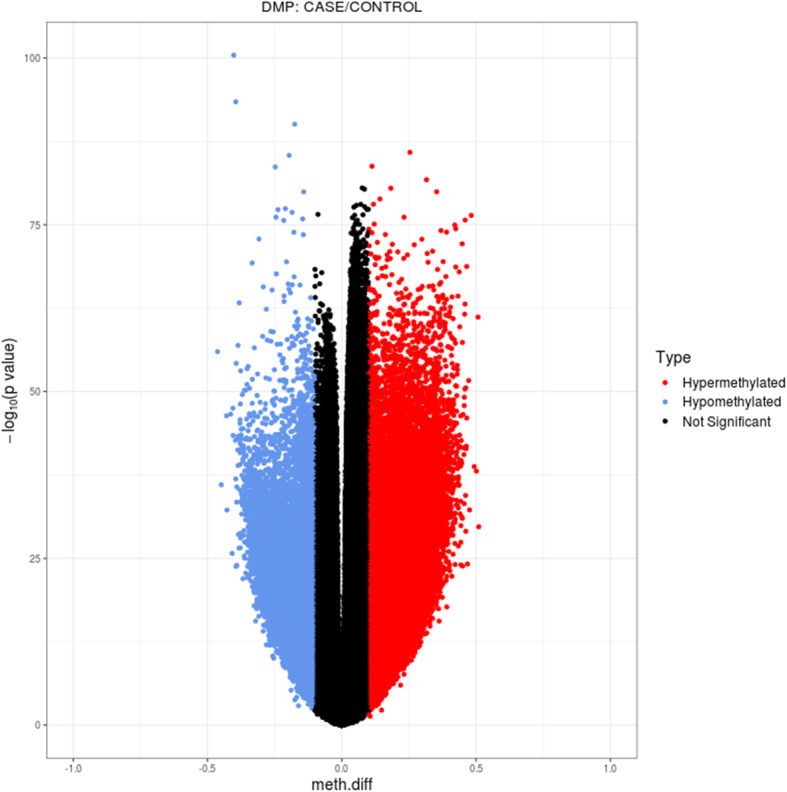
Volcano plot of DMPs. DMPs, differentially methylated positions

**Fig. 3:**
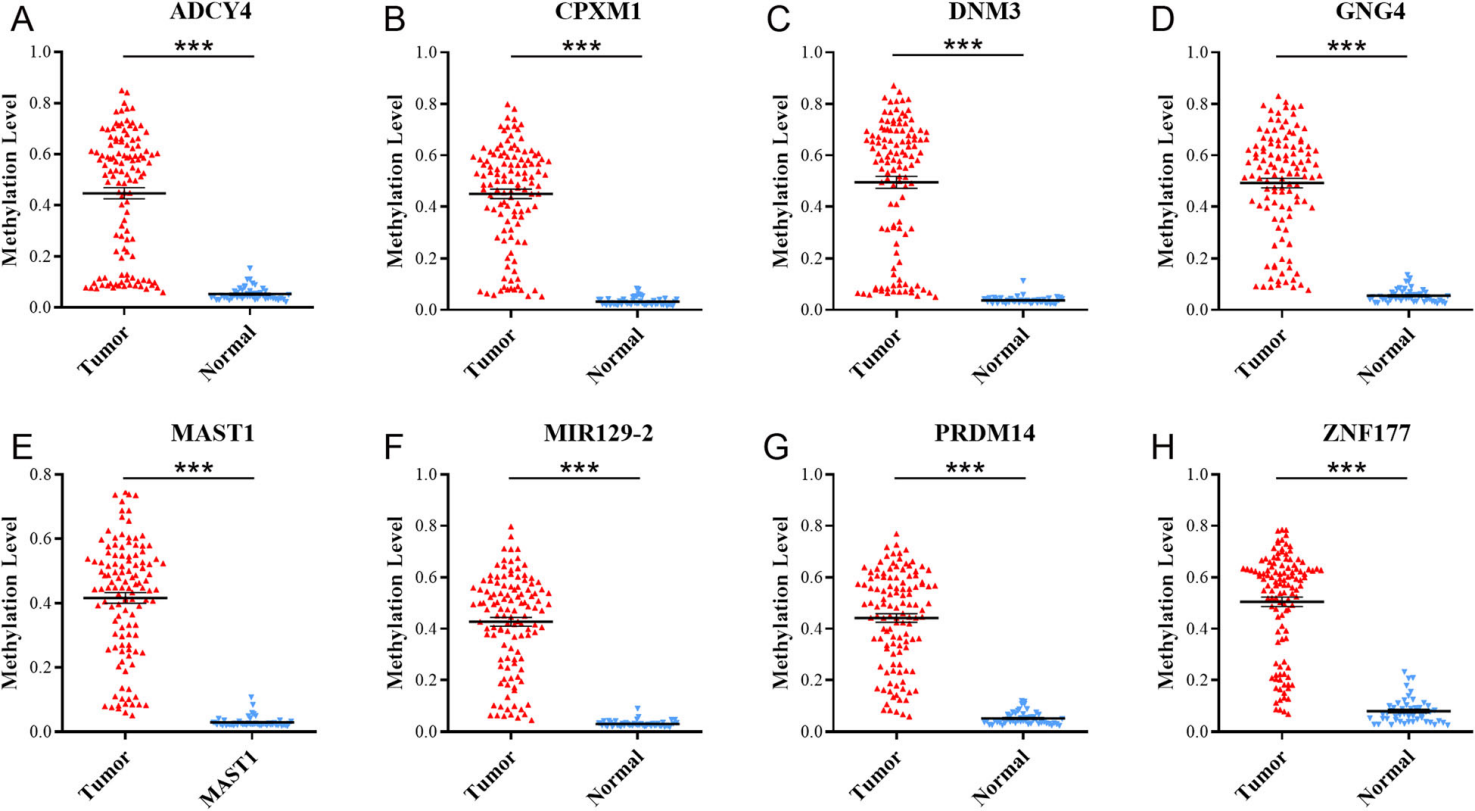
The selected methylation genes had significantly higher levels of DNA methylation in breast cancer as compared with controls. **a** *ADCY4*. **b** *CPXM1*. **c** *DNM3*. **d** *GNG4*. **e** *MAST1*. **f** *mir129-2*. **g** *PRDM14*. **h** *ZNF177*. ***p < 0.001

To evaluate the DNA methylation pattern in breast cancer, we started by comparing 118 breast cancer and 50 control samples. We identified 105,143 differentially methylated positions and 8764 regions in breast cancer cases compared to controls (Fig. 2). Next, we filtered these differentially methylated sites as described in the “Methods” section, allowing us to refine our findings to a total of 23 differentially methylated sites. Importantly, these sites were directly linked to the transcription of 9 genes, namely adenylyl cyclase 4 (*ADCY4*), carboxypeptidase X (*CPXM1*), dynamin 3 (*DNM3*), guanine nucleotide binding-protein gamma subunit 4 (*GNG4*), microtubule associated serine-threonine kinase 1 (*MAST1*), microRNA 129-2 (*mir129*-2), PR domain zinc finger protein 14 (*PRDM14*), protein kinase C beta (*PRKCB*), and zinc finger protein 177 (*ZNF177*) (Table S2; Fig. 3; Table 1). All genes, with exception of *PRKCB*, had significantly higher levels of DNA methylation in breast cancer samples compared to controls.

**Table 1:** Twenty-three differentially methylated sites corresponding to 9 genes

Site	CHR	MAPINFO	Gene	Relation to CpG island	McaM	McoM	*p* value	BH.adjust	DMR
cg27429080	1	171810300	DNM3	N_Shore	0.5053	0.0615	9.76E−25	3.94E−24	DMR_94
cg17154724	1	171810322	DNM3	N_Shore	0.5041	0.0359	7.13E−25	2.90E−24	DMR_94
cg04399751	1	171810376	DNM3	N_Shore	0.4987	0.0487	1.43E−24	5.75E−24	DMR_94
cg04986015	1	171810433	DNM3	N_Shore	0.4563	0.0678	3.25E−25	1.34E−24	DMR_94
cg17816394	1	235814009	GNG4	Island	0.4519	0.0852	6.46E−29	3.12E−28	DMR_179
cg09649610	1	235814039	GNG4	Island	0.5248	0.0813	1.26E−30	6.61E−30	DMR_179
cg00384539	8	70983567	PRDM14	Island	0.4421	0.0848	1.01E−28	4.83E−28	DMR_612
cg13267264	8	70983600	PRDM14	Island	0.4375	0.0826	3.64E−26	1.56E−25	DMR_612
cg14416371	11	43602847	mir129-2	Island	0.4446	0.0395	6.48E−30	3.28E−29	DMR_166
cg01939477	11	43602879	mir129-2	Island	0.4054	0.0260	1.98E−30	1.03E−29	DMR_166
cg16407471	11	43602914	mir129-2	Island	0.4381	0.0688	5.40E−29	2.62E−28	DMR_166
cg05376374	11	43602920	mir129-2	Island	0.4158	0.0452	2.27E−33	1.36E−32	DMR_166
cg23179456	14	24803873	ADCY4	Island	0.4366	0.0623	7.06E−20	2.29E−19	DMR_181
cg12265829	14	24804022	ADCY4	Island	0.4506	0.0883	4.94E−22	1.77E−21	DMR_181
cg03217795	16	23847556	PRKCB	Island	0.4211	0.0246	1.49E−32	8.54E−32	DMR_224
cg05436658	16	23847568	PRKCB	Island	0.3785	0.0173	1.11E−29	5.53E−29	DMR_224
cg08065231	19	9473684	ZNF177	Island	0.4413	0.0755	1.61E−27	7.33E−27	DMR_244
cg09578475	19	9473688	ZNF177	Island	0.5619	0.1547	1.38E−29	6.86E−29	DMR_244
cg08305551	19	12978611	MAST1	Island	0.4241	0.0545	3.83E−30	1.96E−29	DMR_340
cg02776664	19	12978624	MAST1	Island	0.4069	0.0455	4.54E−33	2.68E−32	DMR_340
cg06537894	19	12978706	MAST1	Island	0.4117	0.0575	3.50E−24	1.38E−23	DMR_340
cg22304612	20	2781241	CPXM1	Island	0.4544	0.0626	2.41E−32	1.37E−31	DMR_97
cg07113642	20	2781262	CPXM1	Island	0.4436	0.0294	3.51E−29	1.72E−28	DMR_97

CHR and MAPINFO represent chromosome and position information; McaM represents the mean methylation percentage of the cases, and the McoM represents the mean methylation percentage of the controls; DMR represents differentially methylated region; *p* value is calculated through the Wilcoxon rank-sum test followed by FDR (false discovery rate) adjustment for multiple correction

**Fig. 4:**
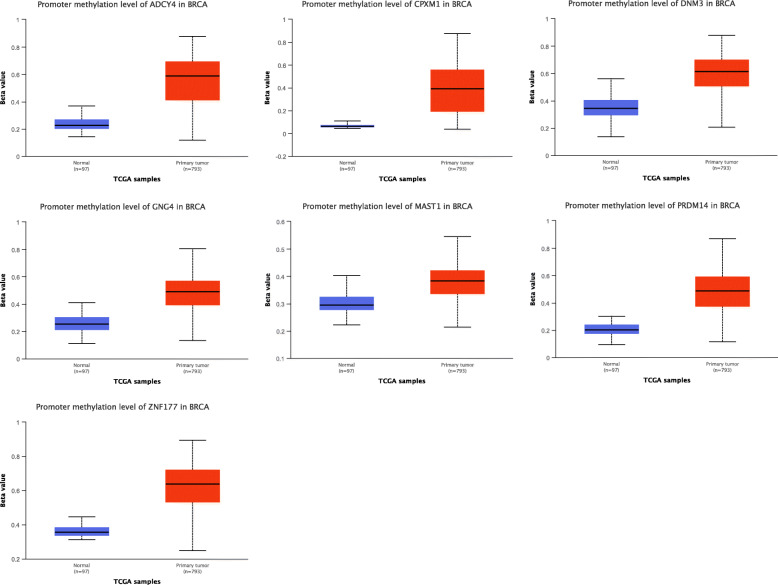
Validation of DNA methylation level using the online tool UALCAN. The methylation levels of **a** *ADCY4*, **b** *CPXM1*, **c** *DNM3*, **d** *GNG4*, **e** *MAST1*, **f** *PRDM14*, and **g** *ZNF177* were also significantly hypermethylated in breast cancer. ***p < 0.001

**Fig. 5:**
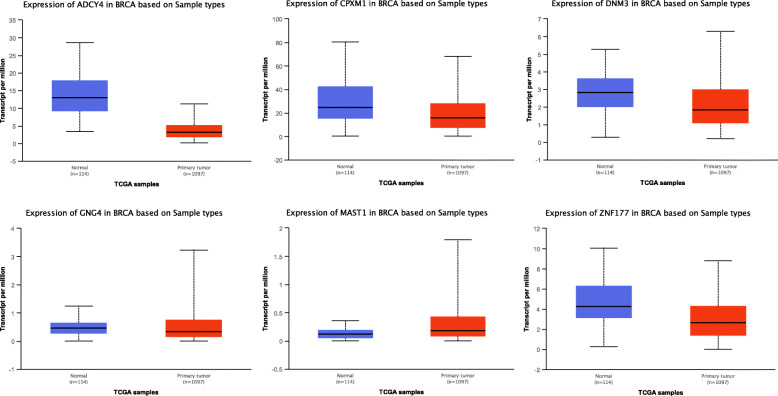
The mRNA expression of selected genes in breast cancer and controls. **a** *ADCY4*. **b** *CPXM1*. **c** *DNM3*. **d** *GNG4*. **e** *MAST1*. **f** *ZNF177*. *p < 0.05; ***p < 0.001

In order to validate the correlation between DNA methylation levels and mRNA expression of the identified genes in breast cancer, we used the online tool UALCAN. As expected, methylation levels of *ADCY4*, *CPXM1*, *DNM3*, *GNG4*, *MAST1*, *PRDM14*, and *ZNF177* were found to be increased in breast cancer, and all with *p* values lower than 0.001 (Fig. 4). Note that information related to *mir129-2* was not available in UALCAN, so we could not conduct this analysis. Then, we found the mRNA expression of *ADCY4*, *CPXM1*, *GNG4*, and *ZNF177* were significantly decreased in breast cancer, the mRNA expression of *MAST1 was* significantly upregulated, but there was no difference of *DNM3* mRNA expression between breast cancer samples and controls (Fig. 5).

### Diagnostic potential of differentially methylated genes

**Fig. 6:**
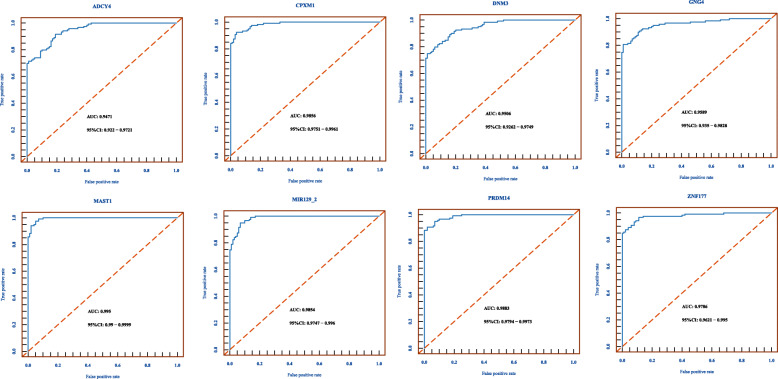
ROC curves and AUC with 95% confidence intervals for the candidate markers. The AUC for individual markers: **a** *ADCY4*, **b** *CPXM1*, **c** *DNM3*, **d** *GNG4*, **e** *MAST1*, **f** *PRDM14*, and **g** *ZNF177*

**Fig. 7:**
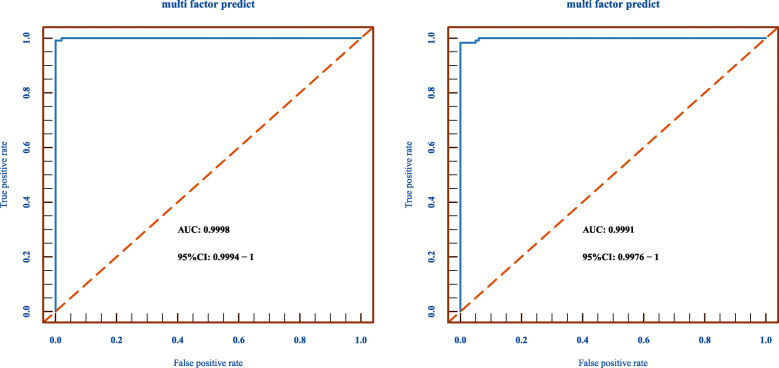
Validating the diagnostic value of the combined logistic regression model in two cohorts. **a** The AUC for the combined signature of 7 genes (*ADCY4*, *CPXM1*, *DNM3*, *GNG4*, *MAST1*, *PRDM14*, *ZNF177*) using a logistic regression model. **b** The AUC for the combined signature of 3 genes (*MAST1*, *PRDM14*, and *ZNF177*) using a logistic regression model

In this study, ROC analyses were performed in GSE72251 and GSE88883 with the pROC package in R Bioconductor to establish thresholds, and only loci showing a threshold below 0.35 were kept. Then, AUC values of *ADCY4* (0.9471), *CPXM1* (0.9856), *DNM3* (0.9506), *GNG4* (0.9589), *MAST1* (0.9950), *PRDM14* (0.9883), and *ZNF177* (0.9786) were all above 0.9 (Fig. 6). Then, we validated the diagnostic value of the combined logistic regression model in these two cohorts, and found the AUC for the combined signature of 7 genes (*ADCY4*, *CPXM1*, *DNM3*, *GNG4*, *MAST1*, *PRDM14*, *ZNF177*) was 0.9998 [95% CI 0.9994–1] (Fig. 7a) and the AUC for the combined signature of 3 genes (*MAST1*, *PRDM14*, and *ZNF177*) was 0.9991 [95% CI 0.9976–1] (Fig. 7b).

Next, each threshold was used to stratify the two mixed cohorts. Our results showed that the breast cancer specificity of each gene ranged from 50.41 to 98.35%, while the sensitivity ranged from 84.25 to 97.64%, and accuracy from 67.82 to 91.13% in mixed cohort 1 (Table 2). Particularly, the specificity, sensitivity, and accuracy of *MAST1* were 81.82%, 97.64%, and 89.92%; those of *PRDM14* were 97.52%, 84.25%, and 90.73%; and those of *ZNF177* were 80.17%, 89.76%, and 85.08%, respectively (Table 2). Results obtained in mixed cohort 2 also followed the same trend, with specificity, sensitivity, and accuracy of *MAST1* being 75.86%, 100%, and 91.95%; of *PRDM14* being 89.66%, 86.21%, and 87.36%; and of *ZNF177* being 89.66%, 93.10%, and 91.95%, respectively (Table 3).

**Table 2:** Validation of 7 differentially methylated genes through mixed cohort 1, including breast cancer (GSE141338, GSE100850, GSE117439) and normal (GSE101961)

Gene	Case, *N*	Control, *N*	Specificity (%)	Sensitivity (%)	Accuracy (%)
ADCY4	81	121	50.41	93.83	67.82
CPXM1	127	121	85.12	95.28	90.32
DNM3	127	121	59.50	89.76	75.00
GNG4	127	121	98.35	84.25	91.13
MAST1	127	121	81.82	97.64	89.92
PRDM14	127	121	97.52	84.25	90.73
ZNF177	127	121	80.17	89.76	85.08

**Table 3:** Validation of 7 differentially methylated genes through mixed cohort 2, including breast cancer (GSE72254) and normal tissues (GSE74214, GSE141338, GSE100850)

Gene	Case, *N*	Control, *N*	Specificity (%)	Sensitivity (%)	Accuracy (%)
ADCY4	58	29	62.07	87.93	79.31
CPXM1	58	29	72.41	79.31	77.01
DNM3	58	29	58.62	62.07	60.92
GNG4	58	29	93.10	63.79	73.56
MAST1	58	29	75.86	100.00	91.95
PRDM14	58	29	89.66	86.21	87.36
ZNF177	58	29	89.66	93.10	91.95

### Prognosis analyzed by K-M plotter

**Fig. 8:**
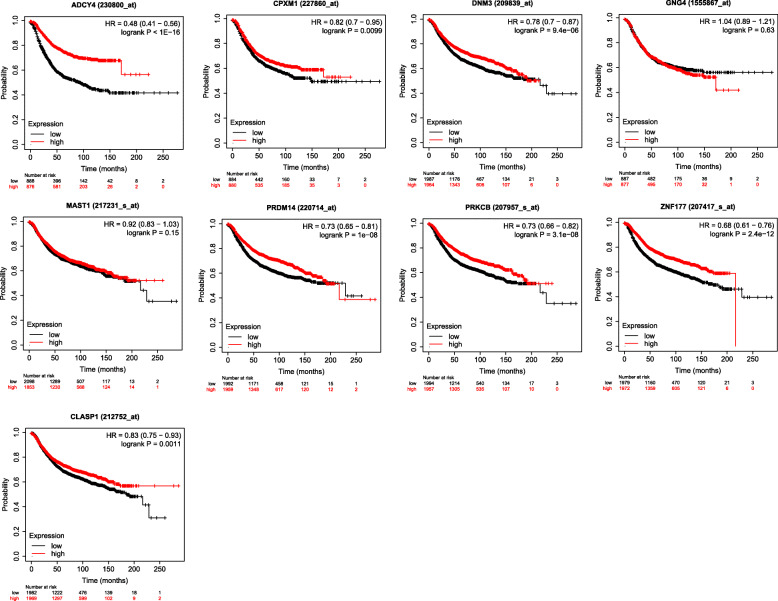
Analysis of differentially methylated genes’ prognosis in breast cancer patients using the Kaplan-Meier plotter. logrank *p* < 0.05 was statistically significant. HR, hazard ratio; the greater the absolute value of (HR-1), the greater the difference between groups in overall survival

To further explore the clinical value of these biomarkers, we evaluated whether 6 of our differentially methylated genes—*ADCY4*, *CPXM1*, *DNM3*, *PRDM14*, *PRKCB*, and *ZNF177*—had any relation with overall survival of breast cancer patients. Hazard ratios of these 6 genes showed significant differences between the high-expression and the low-expression groups, with high expression of all genes being significantly associated with longer overall survival (Fig. 8).

## Discussion

Breast cancer has extremely high mortality worldwide, mostly due to late diagnosis. Cancer-specific DNA methylation patterns are correlated with gene silencing or activation in several types of cancers [19, 20]. Recent studies highlight that aberrant DNA methylation is significantly associated with breast cancer and demonstrated that DNA methylation analysis may help predict the outcome of patients with breast cancer [12, 21]. In this study, we identified differentially methylated genes and confirmed the diagnostic and prognostic value of 6 of these methylation-based biomarkers in breast cancer using a bioinformatics approach.

We first identified 23 differentially methylated CpG sites in breast cancer samples as compared to control counterparts. And the 23 differentially methylated CpG sites correspond to 9 genes, and then, 8 of these 9 genes were coding genes—*ADCY4*, *CPXM1*, *DNM3*, *GNG4*, *MAST1*, *mir129-2*, *PRDM14*, and *ZNF177*—that had significantly higher levels of DNA methylation in breast cancer. Similarly, methylation levels described in UALCAN analysis for all these genes were found to be significantly higher in patients with breast cancer, with exception for *mir129-2* that was not possible to assess. Further analysis revealed the potential of these 8 differentially methylated genes to accurately predict the outcome of patients in training and validation datasets, suggesting that they could be used as biomarkers for breast cancer diagnosis. Additionally, combination of 7 of these methylation markers significantly improved our ability to predict the outcome of breast cancer patients. Overall, we found that *MAST1*, *PRDM14*, and *ZNF177* had high sensitivity, specificity, and accuracy for the diagnosis of breast cancer.

Growing evidence shows a strong relationship between epigenetic and genetic aberrations of *MAST1*, *PRDM14* [22], and *ZNF177* [17] in tumorigenesis. Previous studies reported that abnormal *MAST1* expression is significantly associated with worse cancer prognosis [23, 24]. Oishi et al. found that aberrant promoter demethylation of *MAST1* could be responsible for overexpression of this gene in malignant pheochromocytoma and paraganglioma [25]. Other studies have shown that silencing of *PRDM14* can suppress tumorigenicity and metastasis potential of breast cancer cells [26], while methylation-mediated gene silencing of *PRDM14* leads to apoptosis evasion in human papillomavirus-positive cancer cells [27]. Several reports have also shown that methylation of *ZNF177* is associated with different types of cancer including gastric and endometrial cancers [28], as well as non-small cell lung carcinoma. *ZNF177* is methylation-silenced in gastric cancer cell lines, whereas methylation of its promotor is a frequent epigenetic event in endometrial cancer. Indeed, ROC analysis of *ZNF177* has demonstrated that it can identify endometrial carcinomas cases with a sensitivity, specificity, and accuracy of 92.3%, 94.4%, and 95.1%, respectively. Furthermore, hypermethylated CpG islands within *ZNF177* were selected as candidate biomarker for further validation in NSCLC. Nakakido et al. demonstrated that *ZNF177* is overexpressed in breast cancer and plays a critical role in cancer cell proliferation [29]. However, the role of *MAST1*, *PRDM14*, and *ZNF177* in diagnosis and prognosis of breast cancer remains unclear.

Our findings add a new layer of evidence to the epigenetic landscape of breast cancer, providing convincing clues that *MAST1*, *PRDM14*, and *ZNF177* are differentially methylated in breast cancer, as well as that they may serve as potential drivers and biomarkers for breast cancer. Furthermore, our study demonstrates that high expression of *ADCY4*, *CPXM1*, *DNM3*, *PRDM14*, *PRKCB*, and *ZNF177* are significantly associated with longer patient survival. This finding supports the hypothesis that methylation-driven genes are likely to be associated with clinical outcomes in cancer and can be used as potential biomarkers for predicting the outcome of breast cancer patients.

## Conclusions

In summary, we have identified and independently validated abnormal DNA methylation patterns in *MAST1*, *PRDM14*, and *ZNF177* as potential biomarkers for breast cancer diagnosis. Moreover, we showed that DNA methylation landscape of *ADCY4*, *CPXM1*, *DNM3*, *PRDM14*, *PRKCB*, and *ZNF177* could be selected as accurate biomarkers for the prognosis of breast cancer. Overall, these findings provide a novel epigenetic predictive model that may help improve the diagnosis and prognosis of breast cancer.

## Supplementary Information

**Additional file 1: Table S1.** The information of all samples. **Table S2**. A total of 23 differentially methylated sites were identified.

## Data Availability

The datasets used during the present study are available from the corresponding author upon reasonable request.
