# Supplementary material for: Identification of differentially methylated genes as diagnostic and prognostic biomarkers of breast cancer
Source: World J Surg Oncol. 2021 Jan 26;19:29. doi: 10.1186/s12957-021-02124-6 (PMC7839189; doi:10.1186/s12957-021-02124-6)
Supplement: Supplementary file 1 — Additional file 1: Table S1. The information of all samples. Table S2. A total of 23 differentially methylated sites were identified. [file 12957_2021_2124_MOESM1_ESM.docx]

Supplementary Material

# Supplementary Tables

Table S1. The information of all samples

| project | Sample | description | classify | Sentrix_ID | Sentrix_Position |
| --- | --- | --- | --- | --- | --- |
| GSE72245 | GSM1858429 | Breast tumor from Cohort 1 patient P_1 | case | 5723654034 | R05C01 |
| GSE72245 | GSM1858430 | Breast tumor from Cohort 1 patient P_2 | case | 5723654034 | R02C02 |
| GSE72245 | GSM1858431 | Breast tumor from Cohort 1 patient P_3 | case | 5723654034 | R06C02 |
| GSE72245 | GSM1858432 | Breast tumor from Cohort 1 patient P_4 | case | 5723646043 | R03C02 |
| GSE72245 | GSM1858433 | Breast tumor from Cohort 1 patient P_5 | case | 5723654034 | R06C01 |
| GSE72245 | GSM1858434 | Breast tumor from Cohort 1 patient P_6 | case | 5723654034 | R03C02 |
| GSE72245 | GSM1858435 | Breast tumor from Cohort 1 patient P_7 | case | 5723646043 | R03C01 |
| GSE72245 | GSM1858436 | Breast tumor from Cohort 1 patient P_8 | case | 5723646043 | R01C02 |
| GSE72245 | GSM1858437 | Breast tumor from Cohort 1 patient P_9 | case | 5723654035 | R01C01 |
| GSE72245 | GSM1858438 | Breast tumor from Cohort 1 patient P_10 | case | 5723654035 | R01C02 |
| GSE72245 | GSM1858439 | Breast tumor from Cohort 1 patient P_11 | case | 5723654035 | R02C02 |
| GSE72245 | GSM1858440 | Breast tumor from Cohort 1 patient P_12 | case | 5723654035 | R04C01 |
| GSE72245 | GSM1858441 | Breast tumor from Cohort 1 patient P_13 | case | 5723646043 | R05C01 |
| GSE72245 | GSM1858442 | Breast tumor from Cohort 1 patient P_14 | case | 5723654036 | R01C02 |
| GSE72245 | GSM1858443 | Breast tumor from Cohort 1 patient P_15 | case | 5723654036 | R04C01 |
| GSE72245 | GSM1858444 | Breast tumor from Cohort 1 patient P_16 | case | 5723646043 | R04C01 |
| GSE72245 | GSM1858445 | Breast tumor from Cohort 1 patient P_17 | case | 5723654035 | R05C01 |
| GSE72245 | GSM1858446 | Breast tumor from Cohort 1 patient P_18 | case | 5723654034 | R04C02 |
| GSE72245 | GSM1858447 | Breast tumor from Cohort 1 patient P_19 | case | 5723654034 | R05C02 |
| GSE72245 | GSM1858448 | Breast tumor from Cohort 1 patient P_20 | case | 5723646043 | R05C02 |
| GSE72245 | GSM1858449 | Breast tumor from Cohort 1 patient P_21 | case | 5723646043 | R04C02 |
| GSE72245 | GSM1858450 | Breast tumor from Cohort 1 patient P_22 | case | 5723654036 | R05C01 |
| GSE72245 | GSM1858451 | Breast tumor from Cohort 1 patient P_23 | case | 5723654035 | R06C02 |
| GSE72245 | GSM1858452 | Breast tumor from Cohort 1 patient P_24 | case | 5723654035 | R02C01 |
| GSE72245 | GSM1858453 | Breast tumor from Cohort 1 patient P_25 | case | 5723654048 | R04C01 |
| GSE72245 | GSM1858454 | Breast tumor from Cohort 1 patient P_26 | case | 5723654035 | R04C02 |
| GSE72245 | GSM1858455 | Breast tumor from Cohort 1 patient P_27 | case | 5723654036 | R02C01 |
| GSE72245 | GSM1858456 | Breast tumor from Cohort 1 patient P_28 | case | 5723646043 | R06C01 |
| GSE72245 | GSM1858457 | Breast tumor from Cohort 1 patient P_29 | case | 5723654047 | R02C01 |
| GSE72245 | GSM1858458 | Breast tumor from Cohort 1 patient P_30 | case | 5723654047 | R03C02 |
| GSE72245 | GSM1858459 | Breast tumor from Cohort 1 patient P_31 | case | 5723654035 | R05C02 |
| GSE72245 | GSM1858460 | Breast tumor from Cohort 1 patient P_32 | case | 5723646044 | R01C02 |
| GSE72245 | GSM1858461 | Breast tumor from Cohort 1 patient P_33 | case | 5723654036 | R04C02 |
| GSE72245 | GSM1858462 | Breast tumor from Cohort 1 patient P_34 | case | 5723654036 | R03C02 |
| GSE72245 | GSM1858463 | Breast tumor from Cohort 1 patient P_35 | case | 5723646044 | R05C01 |
| GSE72245 | GSM1858464 | Breast tumor from Cohort 1 patient P_36 | case | 5723654047 | R01C01 |
| GSE72245 | GSM1858465 | Breast tumor from Cohort 1 patient P_37 | case | 5723654047 | R05C02 |
| GSE72245 | GSM1858466 | Breast tumor from Cohort 1 patient P_38 | case | 5723654036 | R03C01 |
| GSE72245 | GSM1858467 | Breast tumor from Cohort 1 patient P_39 | case | 5723646044 | R06C02 |
| GSE72245 | GSM1858468 | Breast tumor from Cohort 1 patient P_40 | case | 5723646043 | R06C02 |
| GSE72245 | GSM1858469 | Breast tumor from Cohort 1 patient P_41 | case | 5723654036 | R05C02 |
| GSE72245 | GSM1858470 | Breast tumor from Cohort 1 patient P_42 | case | 5723646045 | R01C02 |
| GSE72245 | GSM1858471 | Breast tumor from Cohort 1 patient P_43 | case | 5723646044 | R01C01 |
| GSE72245 | GSM1858472 | Breast tumor from Cohort 1 patient P_44 | case | 5723654047 | R06C02 |
| GSE72245 | GSM1858473 | Breast tumor from Cohort 1 patient P_45 | case | 5723646045 | R04C01 |
| GSE72245 | GSM1858474 | Breast tumor from Cohort 1 patient P_46 | case | 5723646044 | R05C02 |
| GSE72245 | GSM1858475 | Breast tumor from Cohort 1 patient P_47 | case | 5723646045 | R06C02 |
| GSE72245 | GSM1858476 | Breast tumor from Cohort 1 patient P_48 | case | 5723646044 | R03C01 |
| GSE72245 | GSM1858477 | Breast tumor from Cohort 1 patient P_49 | case | 5723654036 | R02C02 |
| GSE72245 | GSM1858478 | Breast tumor from Cohort 1 patient P_50 | case | 5723646044 | R04C02 |
| GSE72245 | GSM1858479 | Breast tumor from Cohort 1 patient P_51 | case | 5723646044 | R06C01 |
| GSE72245 | GSM1858480 | Breast tumor from Cohort 1 patient P_52 | case | 5723654036 | R01C01 |
| GSE72245 | GSM1858481 | Breast tumor from Cohort 1 patient P_53 | case | 5723646045 | R01C01 |
| GSE72245 | GSM1858482 | Breast tumor from Cohort 1 patient P_54 | case | 5723646045 | R02C02 |
| GSE72245 | GSM1858483 | Breast tumor from Cohort 1 patient P_55 | case | 5723654047 | R03C01 |
| GSE72245 | GSM1858484 | Breast tumor from Cohort 1 patient P_56 | case | 5723646048 | R01C02 |
| GSE72245 | GSM1858485 | Breast tumor from Cohort 1 patient P_57 | case | 5723654035 | R06C01 |
| GSE72245 | GSM1858486 | Breast tumor from Cohort 1 patient P_58 | case | 5723654047 | R01C02 |
| GSE72245 | GSM1858487 | Breast tumor from Cohort 1 patient P_59 | case | 5723646048 | R03C02 |
| GSE72245 | GSM1858488 | Breast tumor from Cohort 1 patient P_60 | case | 5723646045 | R04C02 |
| GSE72245 | GSM1858489 | Breast tumor from Cohort 1 patient P_61 | case | 5723646043 | R02C01 |
| GSE72245 | GSM1858490 | Breast tumor from Cohort 1 patient P_62 | case | 5723654047 | R05C01 |
| GSE72245 | GSM1858491 | Breast tumor from Cohort 1 patient P_63 | case | 5723654047 | R06C01 |
| GSE72245 | GSM1858492 | Breast tumor from Cohort 1 patient P_64 | case | 5723646048 | R04C01 |
| GSE72245 | GSM1858493 | Breast tumor from Cohort 1 patient P_65 | case | 5723646044 | R03C02 |
| GSE72245 | GSM1858494 | Breast tumor from Cohort 1 patient P_66 | case | 5723654047 | R04C02 |
| GSE72245 | GSM1858495 | Breast tumor from Cohort 1 patient P_67 | case | 5723646048 | R05C01 |
| GSE72245 | GSM1858496 | Breast tumor from Cohort 1 patient P_68 | case | 5723646048 | R06C02 |
| GSE72245 | GSM1858497 | Breast tumor from Cohort 1 patient P_69 | case | 5723654031 | R02C01 |
| GSE72245 | GSM1858498 | Breast tumor from Cohort 1 patient P_70 | case | 5723654031 | R01C02 |
| GSE72245 | GSM1858499 | Breast tumor from Cohort 1 patient P_71 | case | 5723654031 | R05C01 |
| GSE72245 | GSM1858500 | Breast tumor from Cohort 1 patient P_72 | case | 5723654031 | R06C02 |
| GSE72245 | GSM1858501 | Breast tumor from Cohort 1 patient P_73 | case | 5723646045 | R02C01 |
| GSE72245 | GSM1858502 | Breast tumor from Cohort 1 patient P_74 | case | 5723646044 | R04C01 |
| GSE72245 | GSM1858503 | Breast tumor from Cohort 1 patient P_75 | case | 5723654047 | R04C01 |
| GSE72245 | GSM1858504 | Breast tumor from Cohort 1 patient P_76 | case | 5723654031 | R03C02 |
| GSE72245 | GSM1858505 | Breast tumor from Cohort 1 patient P_77 | case | 5723646045 | R05C02 |
| GSE72245 | GSM1858506 | Breast tumor from Cohort 1 patient P_78 | case | 5723646045 | R06C01 |
| GSE72245 | GSM1858507 | Breast tumor from Cohort 1 patient P_79 | case | 5723646045 | R05C01 |
| GSE72245 | GSM1858508 | Breast tumor from Cohort 1 patient P_80 | case | 5723646048 | R01C01 |
| GSE72245 | GSM1858509 | Breast tumor from Cohort 1 patient P_81 | case | 5723654047 | R02C02 |
| GSE72245 | GSM1858510 | Breast tumor from Cohort 1 patient P_82 | case | 5723654032 | R05C02 |
| GSE72245 | GSM1858511 | Breast tumor from Cohort 1 patient P_83 | case | 5723654032 | R01C01 |
| GSE72245 | GSM1858512 | Breast tumor from Cohort 1 patient P_84 | case | 5723654031 | R01C01 |
| GSE72245 | GSM1858513 | Breast tumor from Cohort 1 patient P_85 | case | 5723654032 | R03C01 |
| GSE72245 | GSM1858514 | Breast tumor from Cohort 1 patient P_86 | case | 5723646048 | R03C01 |
| GSE72245 | GSM1858515 | Breast tumor from Cohort 1 patient P_87 | case | 5723646048 | R02C02 |
| GSE72245 | GSM1858516 | Breast tumor from Cohort 1 patient P_88 | case | 5723654032 | R04C02 |
| GSE72245 | GSM1858517 | Breast tumor from Cohort 1 patient P_89 | case | 5723654031 | R03C01 |
| GSE72245 | GSM1858518 | Breast tumor from Cohort 1 patient P_90 | case | 5723654032 | R06C01 |
| GSE72245 | GSM1858519 | Breast tumor from Cohort 1 patient P_91 | case | 5723646048 | R05C02 |
| GSE72245 | GSM1858520 | Breast tumor from Cohort 1 patient P_92 | case | 5723646048 | R06C01 |
| GSE72245 | GSM1858521 | Breast tumor from Cohort 1 patient P_93 | case | 5723654031 | R04C01 |
| GSE72245 | GSM1858522 | Breast tumor from Cohort 1 patient P_94 | case | 5723654031 | R05C02 |
| GSE72245 | GSM1858523 | Breast tumor from Cohort 1 patient P_95 | case | 5723654033 | R01C02 |
| GSE72245 | GSM1858524 | Breast tumor from Cohort 1 patient P_96 | case | 5723654031 | R06C01 |
| GSE72245 | GSM1858525 | Breast tumor from Cohort 1 patient P_97 | case | 5723654032 | R01C02 |
| GSE72245 | GSM1858526 | Breast tumor from Cohort 1 patient P_98 | case | 5723654033 | R04C01 |
| GSE72245 | GSM1858527 | Breast tumor from Cohort 1 patient P_99 | case | 5723654032 | R02C01 |
| GSE72245 | GSM1858528 | Breast tumor from Cohort 1 patient P_100 | case | 5723654032 | R03C02 |
| GSE72245 | GSM1858529 | Breast tumor from Cohort 1 patient P_101 | case | 5723654031 | R04C02 |
| GSE72245 | GSM1858530 | Breast tumor from Cohort 1 patient P_102 | case | 5723654032 | R04C01 |
| GSE72245 | GSM1858531 | Breast tumor from Cohort 1 patient P_103 | case | 5723654033 | R06C01 |
| GSE72245 | GSM1858532 | Breast tumor from Cohort 1 patient P_104 | case | 5723654032 | R05C01 |
| GSE72245 | GSM1858533 | Breast tumor from Cohort 1 patient P_105 | case | 5723654032 | R06C02 |
| GSE72245 | GSM1858534 | Breast tumor from Cohort 1 patient P_106 | case | 5723654048 | R02C02 |
| GSE72245 | GSM1858535 | Breast tumor from Cohort 1 patient P_107 | case | 5723654033 | R02C01 |
| GSE72245 | GSM1858536 | Breast tumor from Cohort 1 patient P_108 | case | 5723654048 | R05C01 |
| GSE72245 | GSM1858537 | Breast tumor from Cohort 1 patient P_109 | case | 5723654033 | R01C01 |
| GSE72245 | GSM1858538 | Breast tumor from Cohort 1 patient P_110 | case | 5723654048 | R03C02 |
| GSE72245 | GSM1858539 | Breast tumor from Cohort 1 patient P_111 | case | 5723654033 | R03C01 |
| GSE72245 | GSM1858540 | Breast tumor from Cohort 1 patient P_112 | case | 5723654033 | R03C02 |
| GSE72245 | GSM1858541 | Breast tumor from Cohort 1 patient P_113 | case | 5723654048 | R06C01 |
| GSE72245 | GSM1858542 | Breast tumor from Cohort 1 patient P_114 | case | 5723654033 | R05C01 |
| GSE72245 | GSM1858543 | Breast tumor from Cohort 1 patient P_115 | case | 5723654048 | R04C02 |
| GSE72245 | GSM1858544 | Breast tumor from Cohort 1 patient P_116 | case | 5723654048 | R01C02 |
| GSE72245 | GSM1858545 | Breast tumor from Cohort 1 patient P_117 | case | 5723654048 | R05C02 |
| GSE72245 | GSM1858546 | Breast tumor from Cohort 1 patient P_118 | case | 5723654048 | R06C02 |
| GSE72251 | GSM1858673 | Breast tumor from Cohort 2 patient P_1 | case | 5723646049 | R02C01 |
| GSE72251 | GSM1858674 | Breast tumor from Cohort 2 patient P_2 | case | 6057833106 | R01C01 |
| GSE72251 | GSM1858675 | Breast tumor from Cohort 2 patient P_3 | case | 6057841036 | R01C02 |
| GSE72251 | GSM1858676 | Breast tumor from Cohort 2 patient P_4 | case | 6057841047 | R03C01 |
| GSE72251 | GSM1858677 | Breast tumor from Cohort 2 patient P_5 | case | 6057841048 | R04C02 |
| GSE72251 | GSM1858678 | Breast tumor from Cohort 2 patient P_6 | case | 6057841131 | R05C01 |
| GSE72251 | GSM1858679 | Breast tumor from Cohort 2 patient P_7 | case | 6057841139 | R06C02 |
| GSE72251 | GSM1858680 | Breast tumor from Cohort 2 patient P_8 | case | 6057841155 | R01C01 |
| GSE72251 | GSM1858681 | Breast tumor from Cohort 2 patient P_9 | case | 6057841061 | R04C02 |
| GSE72251 | GSM1858682 | Breast tumor from Cohort 2 patient P_10 | case | 6057833106 | R03C02 |
| GSE72251 | GSM1858683 | Breast tumor from Cohort 2 patient P_11 | case | 6057841036 | R04C01 |
| GSE72251 | GSM1858684 | Breast tumor from Cohort 2 patient P_12 | case | 6057841047 | R05C02 |
| GSE72251 | GSM1858685 | Breast tumor from Cohort 2 patient P_13 | case | 6057841048 | R02C02 |
| GSE72251 | GSM1858686 | Breast tumor from Cohort 2 patient P_14 | case | 6057841131 | R01C02 |
| GSE72251 | GSM1858687 | Breast tumor from Cohort 2 patient P_15 | case | 6057841139 | R02C01 |
| GSE72251 | GSM1858688 | Breast tumor from Cohort 2 patient P_16 | case | 6057841155 | R03C02 |
| GSE72251 | GSM1858689 | Breast tumor from Cohort 2 patient P_17 | case | 6057841061 | R03C01 |
| GSE72251 | GSM1858690 | Breast tumor from Cohort 2 patient P_18 | case | 6057833106 | R05C02 |
| GSE72251 | GSM1858691 | Breast tumor from Cohort 2 patient P_19 | case | 6057841036 | R06C01 |
| GSE72251 | GSM1858692 | Breast tumor from Cohort 2 patient P_20 | case | 6057841047 | R01C02 |
| GSE72251 | GSM1858693 | Breast tumor from Cohort 2 patient P_21 | case | 6057841048 | R02C01 |
| GSE72251 | GSM1858694 | Breast tumor from Cohort 2 patient P_22 | case | 6057841131 | R03C02 |
| GSE72251 | GSM1858695 | Breast tumor from Cohort 2 patient P_23 | case | 6057841139 | R04C01 |
| GSE72251 | GSM1858696 | Breast tumor from Cohort 2 patient P_24 | case | 6057841155 | R06C02 |
| GSE72251 | GSM1858697 | Breast tumor from Cohort 2 patient P_25 | case | 6057841061 | R05C01 |
| GSE72251 | GSM1858698 | Breast tumor from Cohort 2 patient P_26 | case | 6057833106 | R06C01 |
| GSE72251 | GSM1858699 | Breast tumor from Cohort 2 patient P_27 | case | 6057841139 | R03C02 |
| GSE72251 | GSM1858700 | Breast tumor from Cohort 2 patient P_28 | case | 6057841061 | R05C02 |
| GSE72251 | GSM1858701 | Breast tumor from Cohort 2 patient P_29 | case | 6229068008 | R01C01 |
| GSE72251 | GSM1858702 | Breast tumor from Cohort 2 patient P_30 | case | 6229068008 | R02C01 |
| GSE72251 | GSM1858703 | Breast tumor from Cohort 2 patient P_31 | case | 5723646049 | R02C02 |
| GSE72251 | GSM1858704 | Breast tumor from Cohort 2 patient P_32 | case | 6229068008 | R03C01 |
| GSE72251 | GSM1858705 | Breast tumor from Cohort 2 patient P_33 | case | 6057833106 | R02C01 |
| GSE72251 | GSM1858706 | Breast tumor from Cohort 2 patient P_34 | case | 6057841036 | R02C02 |
| GSE72251 | GSM1858707 | Breast tumor from Cohort 2 patient P_35 | case | 6057841047 | R04C02 |
| GSE72251 | GSM1858708 | Breast tumor from Cohort 2 patient P_36 | case | 6057841048 | R05C01 |
| GSE72251 | GSM1858709 | Breast tumor from Cohort 2 patient P_37 | case | 6057841131 | R02C02 |
| GSE72251 | GSM1858710 | Breast tumor from Cohort 2 patient P_38 | case | 6057841139 | R01C02 |
| GSE72251 | GSM1858711 | Breast tumor from Cohort 2 patient P_39 | case | 6057841155 | R04C02 |
| GSE72251 | GSM1858712 | Breast tumor from Cohort 2 patient P_40 | case | 6057841061 | R06C02 |
| GSE72251 | GSM1858713 | Breast tumor from Cohort 2 patient P_41 | case | 6057833106 | R04C01 |
| GSE72251 | GSM1858714 | Breast tumor from Cohort 2 patient P_42 | case | 6057841036 | R05C01 |
| GSE72251 | GSM1858715 | Breast tumor from Cohort 2 patient P_43 | case | 6057841047 | R06C01 |
| GSE72251 | GSM1858716 | Breast tumor from Cohort 2 patient P_44 | case | 6057841048 | R03C01 |
| GSE72251 | GSM1858717 | Breast tumor from Cohort 2 patient P_45 | case | 6057841131 | R05C02 |
| GSE72251 | GSM1858718 | Breast tumor from Cohort 2 patient P_46 | case | 6229068008 | R04C01 |
| GSE72251 | GSM1858719 | Breast tumor from Cohort 2 patient P_47 | case | 6057841155 | R06C01 |
| GSE72251 | GSM1858720 | Breast tumor from Cohort 2 patient P_48 | case | 6057841061 | R01C01 |
| GSE72251 | GSM1858721 | Breast tumor from Cohort 2 patient P_49 | case | 6057833106 | R06C02 |
| GSE72251 | GSM1858722 | Breast tumor from Cohort 2 patient P_50 | case | 6057841036 | R03C02 |
| GSE72251 | GSM1858723 | Breast tumor from Cohort 2 patient P_51 | case | 6057841047 | R02C01 |
| GSE72251 | GSM1858724 | Breast tumor from Cohort 2 patient P_52 | case | 6057841048 | R01C01 |
| GSE72251 | GSM1858725 | Breast tumor from Cohort 2 patient P_53 | case | 6057841131 | R04C01 |
| GSE72251 | GSM1858726 | Breast tumor from Cohort 2 patient P_54 | case | 6229068008 | R05C01 |
| GSE72251 | GSM1858727 | Breast tumor from Cohort 2 patient P_55 | case | 6057841139 | R05C02 |
| GSE72251 | GSM1858728 | Breast tumor from Cohort 2 patient P_56 | case | 6057841155 | R03C01 |
| GSE72251 | GSM1858729 | Breast tumor from Cohort 2 patient P_57 | case | 6057841061 | R02C02 |
| GSE72251 | GSM1858730 | Breast tumor from Cohort 2 patient P_58 | case | 6057841036 | R06C02 |
| GSE72251 | GSM1858731 | Breast tumor from Cohort 2 patient P_59 | case | 5723646049 | R05C01 |
| GSE72251 | GSM1858732 | Breast tumor from Cohort 2 patient P_60 | case | 6057833106 | R01C02 |
| GSE72251 | GSM1858733 | Breast tumor from Cohort 2 patient P_61 | case | 6229068008 | R06C01 |
| GSE72251 | GSM1858734 | Breast tumor from Cohort 2 patient P_62 | case | 6057833106 | R04C02 |
| GSE72251 | GSM1858735 | Breast tumor from Cohort 2 patient P_63 | case | 6057841036 | R01C01 |
| GSE72251 | GSM1858736 | Breast tumor from Cohort 2 patient P_64 | case | 6057841036 | R03C01 |
| GSE72251 | GSM1858737 | Breast tumor from Cohort 2 patient P_65 | case | 6057841047 | R02C02 |
| GSE72251 | GSM1858738 | Breast tumor from Cohort 2 patient P_66 | case | 6057841047 | R05C01 |
| GSE72251 | GSM1858739 | Breast tumor from Cohort 2 patient P_67 | case | 6057841048 | R04C01 |
| GSE72251 | GSM1858740 | Breast tumor from Cohort 2 patient P_68 | case | 6057841048 | R05C02 |
| GSE72251 | GSM1858741 | Breast tumor from Cohort 2 patient P_69 | case | 6057841048 | R06C01 |
| GSE72251 | GSM1858742 | Breast tumor from Cohort 2 patient P_70 | case | 6057841131 | R02C01 |
| GSE72251 | GSM1858743 | Breast tumor from Cohort 2 patient P_71 | case | 6057841131 | R03C01 |
| GSE72251 | GSM1858744 | Breast tumor from Cohort 2 patient P_72 | case | 6057841131 | R06C02 |
| GSE72251 | GSM1858745 | Breast tumor from Cohort 2 patient P_73 | case | 6057841139 | R01C01 |
| GSE72251 | GSM1858746 | Breast tumor from Cohort 2 patient P_74 | case | 6057841139 | R04C02 |
| GSE72251 | GSM1858747 | Breast tumor from Cohort 2 patient P_75 | case | 6057841139 | R06C01 |
| GSE72251 | GSM1858748 | Breast tumor from Cohort 2 patient P_76 | case | 6057841155 | R01C02 |
| GSE72251 | GSM1858749 | Breast tumor from Cohort 2 patient P_77 | case | 6057841155 | R04C01 |
| GSE72251 | GSM1858750 | Breast tumor from Cohort 2 patient P_78 | case | 6057841155 | R05C01 |
| GSE72251 | GSM1858751 | Breast tumor from Cohort 2 patient P_79 | case | 6057841061 | R02C01 |
| GSE72251 | GSM1858752 | Breast tumor from Cohort 2 patient P_80 | case | 6057841061 | R03C02 |
| GSE72251 | GSM1858753 | Breast tumor from Cohort 2 patient P_81 | case | 6229068022 | R01C02 |
| GSE72251 | GSM1858754 | Breast tumor from Cohort 2 patient P_82 | case | 6229068022 | R02C02 |
| GSE72251 | GSM1858755 | Breast tumor from Cohort 2 patient P_83 | case | 6229068022 | R03C02 |
| GSE72251 | GSM1858756 | Breast tumor from Cohort 2 patient P_84 | case | 5723646049 | R05C02 |
| GSE72251 | GSM1858757 | Breast tumor from Cohort 2 patient P_85 | case | 6229068022 | R04C02 |
| GSE72251 | GSM1858758 | Breast tumor from Cohort 2 patient P_86 | case | 6229068022 | R05C02 |
| GSE72251 | GSM1858759 | Breast tumor from Cohort 2 patient P_87 | case | 6057841131 | R01C01 |
| GSE72251 | GSM1858760 | Breast tumor from Cohort 2 patient P_88 | case | 6057841139 | R03C01 |
| GSE72251 | GSM1858761 | Breast tumor from Cohort 2 patient P_89 | case | 6229068022 | R06C02 |
| GSE72251 | GSM1858762 | Breast tumor from Cohort 2 patient P_90 | case | 6229068025 | R01C01 |
| GSE72251 | GSM1858763 | Breast tumor from Cohort 2 patient P_91 | case | 6057841155 | R02C02 |
| GSE72251 | GSM1858764 | Breast tumor from Cohort 2 patient P_92 | case | 6057841061 | R04C01 |
| GSE72251 | GSM1858765 | Breast tumor from Cohort 2 patient P_93 | case | 6057833106 | R05C01 |
| GSE72251 | GSM1858766 | Breast tumor from Cohort 2 patient P_94 | case | 6057841036 | R04C02 |
| GSE72251 | GSM1858767 | Breast tumor from Cohort 2 patient P_95 | case | 6057841047 | R04C01 |
| GSE72251 | GSM1858768 | Breast tumor from Cohort 2 patient P_96 | case | 6057841048 | R06C02 |
| GSE72251 | GSM1858769 | Breast tumor from Cohort 2 patient P_97 | case | 6229068025 | R02C01 |
| GSE72251 | GSM1858770 | Breast tumor from Cohort 2 patient P_98 | case | 6057841131 | R06C01 |
| GSE72251 | GSM1858771 | Breast tumor from Cohort 2 patient P_99 | case | 6057841139 | R05C01 |
| GSE72251 | GSM1858772 | Breast tumor from Cohort 2 patient P_100 | case | 6057841155 | R05C02 |
| GSE72251 | GSM1858773 | Breast tumor from Cohort 2 patient P_101 | case | 6057841061 | R01C02 |
| GSE72251 | GSM1858774 | Breast tumor from Cohort 2 patient P_102 | case | 6057833106 | R02C02 |
| GSE72251 | GSM1858775 | Breast tumor from Cohort 2 patient P_103 | case | 6057841036 | R02C01 |
| GSE72251 | GSM1858776 | Breast tumor from Cohort 2 patient P_104 | case | 6057841047 | R03C02 |
| GSE72251 | GSM1858777 | Breast tumor from Cohort 2 patient P_105 | case | 6229068025 | R03C01 |
| GSE72251 | GSM1858778 | Breast tumor from Cohort 2 patient P_106 | case | 6057841048 | R03C02 |
| GSE72251 | GSM1858779 | Breast tumor from Cohort 2 patient P_107 | case | 6229041073 | R03C02 |
| GSE72251 | GSM1858780 | Breast tumor from Cohort 2 patient P_108 | case | 6057833106 | R03C01 |
| GSE72251 | GSM1858781 | Breast tumor from Cohort 2 patient P_109 | case | 6057841036 | R05C02 |
| GSE72251 | GSM1858782 | Breast tumor from Cohort 2 patient P_110 | case | 6229041073 | R04C02 |
| GSE72251 | GSM1858783 | Breast tumor from Cohort 2 patient P_111 | case | 6057841047 | R01C01 |
| GSE72251 | GSM1858784 | Breast tumor from Cohort 2 patient P_112 | case | 6057841048 | R01C02 |
| GSE72251 | GSM1858785 | Breast tumor from Cohort 2 patient P_113 | case | 6057841131 | R04C02 |
| GSE72251 | GSM1858786 | Breast tumor from Cohort 2 patient P_114 | case | 6057841139 | R02C02 |
| GSE72251 | GSM1858787 | Breast tumor from Cohort 2 patient P_115 | case | 6057841155 | R02C01 |
| GSE72251 | GSM1858788 | Breast tumor from Cohort 2 patient P_116 | case | 6229041073 | R05C02 |
| GSE72251 | GSM1858789 | Breast tumor from Cohort 2 patient P_117 | case | 6229041073 | R06C02 |
| GSE72251 | GSM1858790 | Breast tumor from Cohort 2 patient P_118 | case | 6057841061 | R06C01 |
| GSE72251 | GSM1858791 | Breast tumor from Cohort 2 patient P_119 | case | 6057841047 | R06C02 |
| GSE72254 | GSM1858792 | Breast tumor from TOP Cohort patient P_1 | case | 6929793128 | R02C02 |
| GSE72254 | GSM1858793 | Breast tumor from TOP Cohort patient P_2 | case | 6929806061 | R01C02 |
| GSE72254 | GSM1858794 | Breast tumor from TOP Cohort patient P_3 | case | 6929742077 | R03C02 |
| GSE72254 | GSM1858795 | Breast tumor from TOP Cohort patient P_4 | case | 6929742020 | R04C01 |
| GSE72254 | GSM1858796 | Breast tumor from TOP Cohort patient P_5 | case | 6929793128 | R05C02 |
| GSE72254 | GSM1858797 | Breast tumor from TOP Cohort patient P_6 | case | 6929793128 | R01C02 |
| GSE72254 | GSM1858798 | Breast tumor from TOP Cohort patient P_7 | case | 6929806021 | R06C02 |
| GSE72254 | GSM1858799 | Breast tumor from TOP Cohort patient P_8 | case | 6929742020 | R01C01 |
| GSE72254 | GSM1858800 | Breast tumor from TOP Cohort patient P_9 | case | 6929742020 | R03C01 |
| GSE72254 | GSM1858801 | Breast tumor from TOP Cohort patient P_10 | case | 6929742020 | R02C02 |
| GSE72254 | GSM1858802 | Breast tumor from TOP Cohort patient P_11 | case | 6929793128 | R04C01 |
| GSE72254 | GSM1858803 | Breast tumor from TOP Cohort patient P_12 | case | 6929793128 | R03C01 |
| GSE72254 | GSM1858804 | Breast tumor from TOP Cohort patient P_13 | case | 6929742077 | R04C01 |
| GSE72254 | GSM1858805 | Breast tumor from TOP Cohort patient P_14 | case | 6929806021 | R03C02 |
| GSE72254 | GSM1858806 | Breast tumor from TOP Cohort patient P_15 | case | 6929742077 | R02C01 |
| GSE72254 | GSM1858807 | Breast tumor from TOP Cohort patient P_16 | case | 6929742020 | R03C02 |
| GSE72254 | GSM1858808 | Breast tumor from TOP Cohort patient P_17 | case | 6929806021 | R03C01 |
| GSE72254 | GSM1858809 | Breast tumor from TOP Cohort patient P_18 | case | 6929742020 | R06C01 |
| GSE72254 | GSM1858810 | Breast tumor from TOP Cohort patient P_19 | case | 6929793128 | R05C01 |
| GSE72254 | GSM1858811 | Breast tumor from TOP Cohort patient P_20 | case | 6929742020 | R01C02 |
| GSE72254 | GSM1858812 | Breast tumor from TOP Cohort patient P_21 | case | 6929742020 | R04C02 |
| GSE72254 | GSM1858813 | Breast tumor from TOP Cohort patient P_22 | case | 6929806021 | R02C01 |
| GSE72254 | GSM1858814 | Breast tumor from TOP Cohort patient P_23 | case | 6929742077 | R05C02 |
| GSE72254 | GSM1858815 | Breast tumor from TOP Cohort patient P_24 | case | 6929793128 | R04C02 |
| GSE72254 | GSM1858816 | Breast tumor from TOP Cohort patient P_25 | case | 6929742020 | R02C01 |
| GSE72254 | GSM1858817 | Breast tumor from TOP Cohort patient P_26 | case | 6929806021 | R01C02 |
| GSE72254 | GSM1858818 | Breast tumor from TOP Cohort patient P_27 | case | 6929806021 | R05C01 |
| GSE72254 | GSM1858819 | Breast tumor from TOP Cohort patient P_28 | case | 6929742077 | R06C02 |
| GSE72254 | GSM1858820 | Breast tumor from TOP Cohort patient P_29 | case | 6929742077 | R03C01 |
| GSE72254 | GSM1858821 | Breast tumor from TOP Cohort patient P_30 | case | 6929806061 | R04C02 |
| GSE72254 | GSM1858822 | Breast tumor from TOP Cohort patient P_31 | case | 6929742077 | R02C02 |
| GSE72254 | GSM1858823 | Breast tumor from TOP Cohort patient P_32 | case | 6929742077 | R04C02 |
| GSE72254 | GSM1858824 | Breast tumor from TOP Cohort patient P_33 | case | 6929742077 | R01C01 |
| GSE72254 | GSM1858825 | Breast tumor from TOP Cohort patient P_34 | case | 6929742077 | R06C01 |
| GSE72254 | GSM1858826 | Breast tumor from TOP Cohort patient P_35 | case | 6929806061 | R06C02 |
| GSE72254 | GSM1858827 | Breast tumor from TOP Cohort patient P_36 | case | 6929742077 | R05C01 |
| GSE72254 | GSM1858828 | Breast tumor from TOP Cohort patient P_37 | case | 6929806021 | R04C02 |
| GSE72254 | GSM1858829 | Breast tumor from TOP Cohort patient P_38 | case | 6929806021 | R05C02 |
| GSE72254 | GSM1858830 | Breast tumor from TOP Cohort patient P_39 | case | 6929806061 | R01C01 |
| GSE72254 | GSM1858831 | Breast tumor from TOP Cohort patient P_40 | case | 6929806061 | R05C02 |
| GSE72254 | GSM1858832 | Breast tumor from TOP Cohort patient P_41 | case | 6929793128 | R01C01 |
| GSE72254 | GSM1858833 | Breast tumor from TOP Cohort patient P_42 | case | 6929742020 | R06C02 |
| GSE72254 | GSM1858834 | Breast tumor from TOP Cohort patient P_43 | case | 6929742077 | R01C02 |
| GSE72254 | GSM1858835 | Breast tumor from TOP Cohort patient P_44 | case | 6929806061 | R04C01 |
| GSE72254 | GSM1858836 | Breast tumor from TOP Cohort patient P_45 | case | 6929793128 | R02C01 |
| GSE72254 | GSM1858837 | Breast tumor from TOP Cohort patient P_46 | case | 6929806061 | R03C01 |
| GSE72254 | GSM1858838 | Breast tumor from TOP Cohort patient P_47 | case | 6929742020 | R05C01 |
| GSE72254 | GSM1858839 | Breast tumor from TOP Cohort patient P_48 | case | 6929742020 | R05C02 |
| GSE72254 | GSM1858840 | Breast tumor from TOP Cohort patient P_49 | case | 6929806021 | R04C01 |
| GSE72254 | GSM1858841 | Breast tumor from TOP Cohort patient P_50 | case | 6929806061 | R02C02 |
| GSE72254 | GSM1858842 | Breast tumor from TOP Cohort patient P_51 | case | 6929806061 | R05C01 |
| GSE72254 | GSM1858843 | Breast tumor from TOP Cohort patient P_52 | case | 6929793128 | R03C02 |
| GSE72254 | GSM1858844 | Breast tumor from TOP Cohort patient P_53 | case | 6929806061 | R03C02 |
| GSE72254 | GSM1858845 | Breast tumor from TOP Cohort patient P_54 | case | 6929806021 | R06C01 |
| GSE72254 | GSM1858846 | Breast tumor from TOP Cohort patient P_55 | case | 6929806061 | R06C01 |
| GSE72254 | GSM1858847 | Breast tumor from TOP Cohort patient P_56 | case | 6929806021 | R01C01 |
| GSE72254 | GSM1858848 | Breast tumor from TOP Cohort patient P_57 | case | 6929806021 | R02C02 |
| GSE72254 | GSM1858849 | Breast tumor from TOP Cohort patient P_58 | case | 6929806061 | R02C01 |
| GSE88883 | GSM2350679 | K102424 | control | 3999941156 | R01C01 |
| GSE88883 | GSM2350680 | K102434 | control | 3999932019 | R01C01 |
| GSE88883 | GSM2350681 | K102440 | control | 3999941118 | R05C01 |
| GSE88883 | GSM2350682 | K102448 | control | 3999941159 | R01C01 |
| GSE88883 | GSM2350683 | K102449 | control | 3999932028 | R01C01 |
| GSE88883 | GSM2350684 | K102457 | control | 3999932027 | R01C01 |
| GSE88883 | GSM2350685 | K102460 | control | 3999932057 | R01C01 |
| GSE88883 | GSM2350686 | K102469 | control | 3999932049 | R01C01 |
| GSE88883 | GSM2350687 | K102475 | control | 3999932055 | R01C01 |
| GSE88883 | GSM2350688 | K102490 | control | 3999941156 | R02C01 |
| GSE88883 | GSM2350689 | K102491 | control | 3999932019 | R02C01 |
| GSE88883 | GSM2350690 | K102499 | control | 3999941159 | R05C02 |
| GSE88883 | GSM2350691 | K102500 | control | 3999941159 | R02C01 |
| GSE88883 | GSM2350692 | K102503 | control | 3999932028 | R02C01 |
| GSE88883 | GSM2350693 | K102511 | control | 3999932027 | R02C01 |
| GSE88883 | GSM2350694 | K102517 | control | 3999932057 | R02C01 |
| GSE88883 | GSM2350695 | K102522 | control | 3999932049 | R02C01 |
| GSE88883 | GSM2350696 | K102524 | control | 3999932055 | R02C01 |
| GSE88883 | GSM2350697 | K102529 | control | 3999941156 | R03C01 |
| GSE88883 | GSM2350698 | K102530 | control | 3999932019 | R03C01 |
| GSE88883 | GSM2350699 | K102531 | control | 3999941159 | R03C01 |
| GSE88883 | GSM2350700 | K102535 | control | 3999932028 | R03C01 |
| GSE88883 | GSM2350701 | K102536 | control | 3999932027 | R03C01 |
| GSE88883 | GSM2350702 | K102546 | control | 3999932057 | R03C01 |
| GSE88883 | GSM2350703 | K102547 | control | 3999932049 | R03C01 |
| GSE88883 | GSM2350704 | K102559 | control | 3999932055 | R03C01 |
| GSE88883 | GSM2350705 | K102572 | control | 3999941156 | R04C01 |
| GSE88883 | GSM2350706 | K102573 | control | 3999932019 | R04C01 |
| GSE88883 | GSM2350707 | K102574 | control | 3999941159 | R04C01 |
| GSE88883 | GSM2350708 | K102575 | control | 3999932028 | R05C02 |
| GSE88883 | GSM2350709 | K102590 | control | 3999932028 | R04C01 |
| GSE88883 | GSM2350710 | K102591 | control | 3999932027 | R04C01 |
| GSE88883 | GSM2350711 | K102602 | control | 3999932057 | R04C01 |
| GSE88883 | GSM2350712 | K102609 | control | 3999932027 | R05C02 |
| GSE88883 | GSM2350713 | K102623 | control | 3999932057 | R05C02 |
| GSE88883 | GSM2350714 | K102625 | control | 3999941118 | R04C01 |
| GSE88883 | GSM2350715 | K102629 | control | 3999932049 | R05C02 |
| GSE88883 | GSM2350716 | K102639 | control | 3999932055 | R05C02 |
| GSE88883 | GSM2350717 | K102664 | control | 3999941156 | R06C02 |
| GSE88883 | GSM2350718 | K102669 | control | 3999932049 | R04C01 |
| GSE88883 | GSM2350719 | K102675 | control | 3999932019 | R06C02 |
| GSE88883 | GSM2350720 | K102685 | control | 3999941159 | R06C02 |
| GSE88883 | GSM2350721 | K102686 | control | 3999932028 | R06C02 |
| GSE88883 | GSM2350722 | K102690 | control | 3999932027 | R06C02 |
| GSE88883 | GSM2350723 | K103103 | control | 3999932055 | R04C01 |
| GSE88883 | GSM2350724 | K103104 | control | 3999941156 | R05C01 |
| GSE88883 | GSM2350725 | K104153 | control | 3999932019 | R05C01 |
| GSE88883 | GSM2350726 | K104157 | control | 3999941159 | R05C01 |
| GSE88883 | GSM2350727 | K104158 | control | 3999932028 | R05C01 |
| GSE88883 | GSM2350728 | K104159 | control | 3999932027 | R05C01 |
| GSE88883 | GSM2350729 | K104162 | control | 3999932057 | R05C01 |
| GSE88883 | GSM2350730 | K104167 | control | 3999932049 | R05C01 |
| GSE88883 | GSM2350731 | K104173 | control | 3999932055 | R05C01 |
| GSE88883 | GSM2350732 | K104174 | control | 3999941156 | R06C01 |
| GSE88883 | GSM2350733 | K104178 | control | 3999932019 | R06C01 |
| GSE88883 | GSM2350734 | K104182 | control | 3999941159 | R06C01 |
| GSE88883 | GSM2350735 | K104193 | control | 3999932028 | R06C01 |
| GSE88883 | GSM2350736 | K104196 | control | 3999932027 | R06C01 |
| GSE88883 | GSM2350737 | K104204 | control | 3999932057 | R06C01 |
| GSE88883 | GSM2350738 | K104205 | control | 3999932049 | R06C01 |
| GSE88883 | GSM2350739 | K104210 | control | 3999932055 | R06C01 |
| GSE88883 | GSM2350740 | K104214 | control | 3999941156 | R01C02 |
| GSE88883 | GSM2350741 | K104221 | control | 3999932019 | R01C02 |
| GSE88883 | GSM2350742 | K104249 | control | 3999932057 | R06C02 |
| GSE88883 | GSM2350743 | K104252 | control | 3999941159 | R01C02 |
| GSE88883 | GSM2350744 | K104260 | control | 3999932028 | R01C02 |
| GSE88883 | GSM2350745 | K104261 | control | 3999932027 | R01C02 |
| GSE88883 | GSM2350746 | K104263 | control | 3999941118 | R01C02 |
| GSE88883 | GSM2350747 | K104268 | control | 3999932057 | R01C02 |
| GSE88883 | GSM2350748 | K104270 | control | 3999932049 | R01C02 |
| GSE88883 | GSM2350749 | K104273 | control | 3999932055 | R01C02 |
| GSE88883 | GSM2350750 | K104276 | control | 3999941156 | R02C02 |
| GSE88883 | GSM2350751 | K104280 | control | 3999932019 | R02C02 |
| GSE88883 | GSM2350752 | K104281 | control | 3999941159 | R02C02 |
| GSE88883 | GSM2350753 | K104283 | control | 3999932028 | R02C02 |
| GSE88883 | GSM2350754 | K104285 | control | 3999932027 | R02C02 |
| GSE88883 | GSM2350755 | K104286 | control | 3999932057 | R02C02 |
| GSE88883 | GSM2350756 | K104295 | control | 3999932049 | R02C02 |
| GSE88883 | GSM2350757 | K104296 | control | 3999932055 | R02C02 |
| GSE88883 | GSM2350758 | K104298 | control | 3999941156 | R03C02 |
| GSE88883 | GSM2350759 | K104304 | control | 3999932019 | R03C02 |
| GSE88883 | GSM2350760 | K104307 | control | 3999941159 | R03C02 |
| GSE88883 | GSM2350761 | K104313 | control | 3999941118 | R05C02 |
| GSE88883 | GSM2350762 | K104332 | control | 3999932028 | R03C02 |
| GSE88883 | GSM2350763 | K104348 | control | 3999932027 | R03C02 |
| GSE88883 | GSM2350764 | K104352 | control | 3999932057 | R03C02 |
| GSE88883 | GSM2350765 | K104359 | control | 3999932049 | R03C02 |
| GSE88883 | GSM2350766 | K104364 | control | 3999932055 | R03C02 |
| GSE88883 | GSM2350767 | K104365 | control | 3999941156 | R04C02 |
| GSE88883 | GSM2350768 | K104368 | control | 3999932019 | R04C02 |
| GSE88883 | GSM2350769 | K104370 | control | 3999941159 | R04C02 |
| GSE88883 | GSM2350770 | K104377 | control | 3999932028 | R04C02 |
| GSE88883 | GSM2350771 | K104388 | control | 3999932027 | R04C02 |
| GSE88883 | GSM2350772 | K104397 | control | 3999932057 | R04C02 |
| GSE88883 | GSM2350773 | K104398 | control | 3999932049 | R04C02 |
| GSE88883 | GSM2350774 | K104402 | control | 3999932049 | R06C02 |
| GSE88883 | GSM2350775 | K104489 | control | 3999932055 | R04C02 |
| GSE88883 | GSM2350776 | K104495 | control | 3999941156 | R05C02 |
| GSE88883 | GSM2350777 | K104766 | control | 3999932019 | R05C02 |
| GSE88883 | GSM2350778 | K105115 | control | 3999932055 | R06C02 |
| GSE74214 | GSM1914651 | breast_tissue_1_BS | control | 3999876061 | R04C01 |
| GSE74214 | GSM1914653 | breast_tissue_2_BS | control | 3999876061 | R05C02 |
| GSE74214 | GSM1914655 | breast_tissue_3_BS | control | 3999876061 | R06C02 |
| GSE74214 | GSM1914657 | breast_tissue_4_BS | control | 3999876061 | R04C02 |
| GSE74214 | GSM1914659 | breast_tissue_5_BS | control | 3999876061 | R01C01 |
| GSE74214 | GSM1914661 | breast_tissue_6_BS | control | 3999876061 | R03C02 |
| GSE74214 | GSM1914663 | breast_tissue_7_BS | control | 3999876016 | R02C01 |
| GSE74214 | GSM1914665 | breast_tissue_8_BS | control | 3999876016 | R06C01 |
| GSE74214 | GSM1914667 | breast_tissue_9_BS | control | 3999876016 | R04C02 |
| GSE74214 | GSM1914669 | breast_tissue_10_BS | control | 3999876016 | R05C01 |
| GSE74214 | GSM1914671 | breast_tissue_11_BS | control | 3999876016 | R02C02 |
| GSE74214 | GSM1914673 | breast_tissue_12_BS | control | 3999876016 | R04C01 |
| GSE74214 | GSM1914675 | breast_tissue_13_BS | control | 3999876017 | R02C02 |
| GSE74214 | GSM1914677 | breast_tissue_14_BS | control | 3999876017 | R01C01 |
| GSE74214 | GSM1914679 | breast_tissue_15_BS | control | 3999876017 | R04C01 |
| GSE74214 | GSM1914681 | breast_tissue_16_BS | control | 3999876017 | R06C01 |
| GSE74214 | GSM1914683 | breast_tissue_17_BS | control | 3999876017 | R02C01 |
| GSE74214 | GSM1914685 | breast_tissue_18_BS | control | 3999876017 | R04C02 |
| GSE141338 | GSM4201284 | genomic DNA from normal breast tissue 1 | control | 9992576115 | R01C01 |
| GSE141338 | GSM4201285 | genomic DNA from normal breast tissue 2 | control | 9992576115 | R05C01 |
| GSE141338 | GSM4201286 | genomic DNA from normal breast tissue 3 | control | 9992576115 | R03C02 |
| GSE141338 | GSM4201287 | genomic DNA from normal breast tissue 4 | control | 9992576144 | R01C01 |
| GSE141338 | GSM4201288 | genomic DNA from normal breast tissue 5 | control | 9992576144 | R05C01 |
| GSE141338 | GSM4201289 | genomic DNA from normal breast tissue 6 | control | 9992576144 | R03C02 |
| GSE141338 | GSM4201290 | genomic DNA from luminal A tumor (case 1) | case | 9992576148 | R01C01 |
| GSE141338 | GSM4201291 | genomic DNA from luminal A tumor (case 2) | case | 9992576148 | R05C01 |
| GSE141338 | GSM4201292 | genomic DNA from luminal A tumor (case 3) | case | 9992576148 | R03C02 |
| GSE141338 | GSM4201293 | genomic DNA from luminal A tumor (case 4) | case | 9992576158 | R01C01 |
| GSE141338 | GSM4201294 | genomic DNA from luminal A tumor (case 5) | case | 9992576158 | R05C01 |
| GSE141338 | GSM4201295 | genomic DNA from luminal A tumor (case 6) | case | 9992576158 | R02C02 |
| GSE141338 | GSM4201296 | genomic DNA from luminal A tumor (case 7) | case | 9992576115 | R02C01 |
| GSE141338 | GSM4201297 | genomic DNA from luminal A tumor (case 8) | case | 9992576115 | R06C01 |
| GSE141338 | GSM4201298 | genomic DNA from luminal B tumor (case 1) | case | 9992576115 | R04C02 |
| GSE141338 | GSM4201299 | genomic DNA from luminal B tumor (case 2) | case | 9992576144 | R02C01 |
| GSE141338 | GSM4201300 | genomic DNA from luminal B tumor (case 3) | case | 9992576144 | R06C01 |
| GSE141338 | GSM4201301 | genomic DNA from luminal B tumor (case 4) | case | 9992576144 | R04C02 |
| GSE141338 | GSM4201302 | genomic DNA from luminal B tumor (case 5) | case | 9992576148 | R02C01 |
| GSE141338 | GSM4201303 | genomic DNA from luminal B tumor (case 6) | case | 9992576148 | R06C01 |
| GSE141338 | GSM4201304 | genomic DNA from luminal B tumor (case 7) | case | 9992576148 | R04C02 |
| GSE141338 | GSM4201305 | genomic DNA from luminal B tumor (case 8) | case | 9992576158 | R02C01 |
| GSE141338 | GSM4201306 | genomic DNA from luminal-HER2 tumor (case 1) | case | 9992576158 | R06C01 |
| GSE141338 | GSM4201307 | genomic DNA from luminal-HER2 tumor (case 2) | case | 9992576158 | R03C02 |
| GSE141338 | GSM4201308 | genomic DNA from luminal-HER2 tumor (case 3) | case | 9992576115 | R03C01 |
| GSE141338 | GSM4201309 | genomic DNA from luminal-HER2 tumor (case 4) | case | 9992576115 | R01C02 |
| GSE141338 | GSM4201310 | genomic DNA from luminal-HER2 tumor (case 5) | case | 9992576115 | R05C02 |
| GSE141338 | GSM4201311 | genomic DNA from luminal-HER2 tumor (case 6) | case | 9992576144 | R03C01 |
| GSE141338 | GSM4201312 | genomic DNA from luminal-HER2 tumor (case 7) | case | 9992576144 | R01C02 |
| GSE141338 | GSM4201313 | genomic DNA from luminal-HER2 tumor (case 8) | case | 9992576144 | R05C02 |
| GSE141338 | GSM4201314 | genomic DNA from HER2 tumor (case 1) | case | 9992576148 | R03C01 |
| GSE141338 | GSM4201315 | genomic DNA from HER2 tumor (case 2) | case | 9992576148 | R01C02 |
| GSE141338 | GSM4201316 | genomic DNA from HER2 tumor (case 3) | case | 9992576148 | R05C02 |
| GSE141338 | GSM4201317 | genomic DNA from HER2 tumor (case 4) | case | 9992576158 | R03C01 |
| GSE141338 | GSM4201318 | genomic DNA from HER2 tumor (case 5) | case | 9992576158 | R01C02 |
| GSE141338 | GSM4201319 | genomic DNA from HER2 tumor (case 6) | case | 9992576158 | R04C02 |
| GSE141338 | GSM4201320 | genomic DNA from HER2 tumor (case 7) | case | 9992576115 | R04C01 |
| GSE141338 | GSM4201321 | genomic DNA from HER2 tumor (case 8) | case | 9992576115 | R02C02 |
| GSE141338 | GSM4201322 | genomic DNA from TN tumor (case 1) | case | 9992576115 | R06C02 |
| GSE141338 | GSM4201323 | genomic DNA from TN tumor (case 2) | case | 9992576144 | R04C01 |
| GSE141338 | GSM4201324 | genomic DNA from TN tumor (case 3) | case | 9992576144 | R02C02 |
| GSE141338 | GSM4201325 | genomic DNA from TN tumor (case 4) | case | 9992576144 | R06C02 |
| GSE141338 | GSM4201326 | genomic DNA from TN tumor (case 5) | case | 9992576148 | R04C01 |
| GSE141338 | GSM4201327 | genomic DNA from TN tumor (case 6) | case | 9992576148 | R02C02 |
| GSE141338 | GSM4201328 | genomic DNA from TN tumor (case 7) | case | 9992576148 | R06C02 |
| GSE141338 | GSM4201329 | genomic DNA from TN tumor (case 8) | case | 9992576158 | R04C01 |
| GSE141338 | GSM4201330 | genomic DNA from luminal A tumor (case 9) | case | 9992576158 | R05C02 |
| GSE141338 | GSM4201331 | in vitro methylated DNA | other | 9992576158 | R06C02 |
| GSE100850 | GSM2695082 | Normal Breast tissue_Old (200526210010_R01C01) | control | 200526210010 | R01C01 |
| GSE100850 | GSM2695083 | Breast Cancer_Old (200526210010_R02C01) | case | 200526210010 | R02C01 |
| GSE100850 | GSM2695084 | Breast Cancer_Old (200526210010_R03C01) | case | 200526210010 | R03C01 |
| GSE100850 | GSM2695085 | Breast Cancer_Young (200526210010_R06C01) | case | 200526210010 | R06C01 |
| GSE100850 | GSM2695086 | Breast Cancer_Old (200526210010_R07C01) | case | 200526210010 | R07C01 |
| GSE100850 | GSM2695087 | Breast Cancer_Young (200526210010_R08C01) | case | 200526210010 | R08C01 |
| GSE100850 | GSM2695088 | Breast Cancer_Old (200526210139_R01C01) | case | 200526210139 | R01C01 |
| GSE100850 | GSM2695089 | Breast Cancer_Young (200526210139_R02C01) | case | 200526210139 | R02C01 |
| GSE100850 | GSM2695090 | Normal Breast tissue_Young (200526210139_R03C01) | control | 200526210139 | R03C01 |
| GSE100850 | GSM2695091 | Breast Cancer_Young (200526210139_R04C01) | case | 200526210139 | R04C01 |
| GSE100850 | GSM2695092 | Breast Cancer_Old (200526210139_R05C01) | case | 200526210139 | R05C01 |
| GSE100850 | GSM2695093 | Breast Cancer_Young (200526210139_R06C01) | case | 200526210139 | R06C01 |
| GSE100850 | GSM2695094 | Breast Cancer_Old (200526210139_R07C01) | case | 200526210139 | R07C01 |
| GSE100850 | GSM2695095 | Breast Cancer_Young (200526210139_R08C01) | case | 200526210139 | R08C01 |
| GSE100850 | GSM2695096 | Breast Cancer_Young (200526210187_R01C01) | case | 200526210187 | R01C01 |
| GSE100850 | GSM2695097 | Normal Breast tissue_Old (200526210187_R02C01) | control | 200526210187 | R02C01 |
| GSE100850 | GSM2695098 | Breast Cancer_Young (200526210187_R03C01) | case | 200526210187 | R03C01 |
| GSE100850 | GSM2695099 | Breast Cancer_Old (200526210187_R04C01) | case | 200526210187 | R04C01 |
| GSE100850 | GSM2695100 | Breast Cancer_Old (200526210187_R06C01) | case | 200526210187 | R06C01 |
| GSE100850 | GSM2695101 | Breast Cancer_Young (200526210187_R07C01) | case | 200526210187 | R07C01 |
| GSE100850 | GSM2695102 | Breast Cancer_Old (200526210187_R08C01) | case | 200526210187 | R08C01 |
| GSE100850 | GSM2695103 | Breast Cancer_Young (200526210135_R02C01) | case | 200526210135 | R02C01 |
| GSE100850 | GSM2695104 | Breast Cancer_Old (200526210135_R03C01) | case | 200526210135 | R03C01 |
| GSE100850 | GSM2695105 | Normal Breast tissue_Old (200526210135_R04C01) | control | 200526210135 | R04C01 |
| GSE100850 | GSM2695106 | Breast Cancer_Old (200526210135_R05C01) | case | 200526210135 | R05C01 |
| GSE100850 | GSM2695107 | Breast Cancer_Young (200526210135_R06C01) | case | 200526210135 | R06C01 |
| GSE100850 | GSM2695108 | Breast Cancer_Young (200526210135_R07C01) | case | 200526210135 | R07C01 |
| GSE100850 | GSM2695109 | Breast Cancer_Young (200526210135_R08C01) | case | 200526210135 | R08C01 |
| GSE100850 | GSM2695110 | Breast Cancer_Young (200526210006_R01C01) | case | 200526210006 | R01C01 |
| GSE100850 | GSM2695111 | Breast Cancer_Young (200526210006_R02C01) | case | 200526210006 | R02C01 |
| GSE100850 | GSM2695112 | Breast Cancer_Young (200526210006_R03C01) | case | 200526210006 | R03C01 |
| GSE100850 | GSM2695113 | Breast Cancer_Young (200526210006_R04C01) | case | 200526210006 | R04C01 |
| GSE100850 | GSM2695114 | Normal Breast tissue_Young (200526210006_R05C01) | control | 200526210006 | R05C01 |
| GSE100850 | GSM2695115 | Breast Cancer_Old (200526210006_R06C01) | case | 200526210006 | R06C01 |
| GSE100850 | GSM2695116 | Breast Cancer_Young (200526210052_R01C01) | case | 200526210052 | R01C01 |
| GSE100850 | GSM2695117 | Breast Cancer_Young (200526210052_R04C01) | case | 200526210052 | R04C01 |
| GSE100850 | GSM2695118 | Breast Cancer_Young (200526210052_R05C01) | case | 200526210052 | R05C01 |
| GSE100850 | GSM2695119 | Breast Cancer_Old (200526210052_R07C01) | case | 200526210052 | R07C01 |
| GSE100850 | GSM2695120 | Breast Cancer_Young (200526210052_R08C01) | case | 200526210052 | R08C01 |
| GSE117439 | GSM3294759 | sample 1, primary tumor from ERpos BC | case | 9422492137 | R02C02 |
| GSE117439 | GSM3294760 | sample 1, second tumor from ERpos BC | case | 9422492137 | R03C02 |
| GSE117439 | GSM3294761 | sample 2, primary tumor from ERpos BC | case | 9422492063 | R01C02 |
| GSE117439 | GSM3294762 | sample 2, second tumor from ERpos BC | case | 9422492082 | R02C01 |
| GSE117439 | GSM3294763 | sample 3, primary tumor from ERpos BC | case | 9422492082 | R03C01 |
| GSE117439 | GSM3294764 | sample 3, second tumor from ERpos BC | case | 9422492063 | R04C02 |
| GSE117439 | GSM3294765 | sample 4, primary tumor from ERpos BC | case | 9421912123 | R04C02 |
| GSE117439 | GSM3294766 | sample 4, second tumor from ERpos BC | case | 9422491157 | R02C01 |
| GSE117439 | GSM3294767 | sample 5, primary tumor from ERpos BC | case | 9422492137 | R01C01 |
| GSE117439 | GSM3294768 | sample 5, second tumor from ERpos BC | case | 9421912123 | R03C02 |
| GSE117439 | GSM3294769 | sample 6, primary tumor from ERpos BC | case | 9422492082 | R06C02 |
| GSE117439 | GSM3294770 | sample 6, second tumor from ERpos BC | case | 9422492063 | R03C02 |
| GSE117439 | GSM3294771 | sample 7, primary tumor from ERpos BC | case | 9422492063 | R02C02 |
| GSE117439 | GSM3294772 | sample 7, second tumor from ERpos BC | case | 9422492137 | R04C02 |
| GSE117439 | GSM3294773 | sample 8, primary tumor from ERpos BC | case | 9422491156 | R04C01 |
| GSE117439 | GSM3294774 | sample 8, second tumor from ERpos BC | case | 9422492063 | R05C01 |
| GSE117439 | GSM3294775 | sample 9, primary tumor from ERpos BC | case | 9422491156 | R06C02 |
| GSE117439 | GSM3294776 | sample 9, second tumor from ERpos BC | case | 9422491157 | R05C01 |
| GSE117439 | GSM3294777 | sample 10, primary tumor from ERpos BC | case | 9422491156 | R06C01 |
| GSE117439 | GSM3294778 | sample 10, second tumor from ERpos BC | case | 9422491156 | R03C02 |
| GSE117439 | GSM3294779 | sample 11, primary tumor from ERpos BC | case | 9422492063 | R06C01 |
| GSE117439 | GSM3294780 | sample 11, second tumor from ERpos BC | case | 9422492137 | R01C02 |
| GSE117439 | GSM3294781 | sample 12, primary tumor from ERpos BC | case | 9422492063 | R03C01 |
| GSE117439 | GSM3294782 | sample 12, second tumor from ERpos BC | case | 9422492063 | R02C01 |
| GSE117439 | GSM3294783 | sample 13, primary tumor from ERneg BC | case | 9422491156 | R03C01 |
| GSE117439 | GSM3294784 | sample 13, second tumor from ERneg BC | case | 9422491157 | R01C02 |
| GSE117439 | GSM3294785 | sample 14, primary tumor from ERneg BC | case | 9422491156 | R02C02 |
| GSE117439 | GSM3294786 | sample 14, second tumor from ERneg BC | case | 9422491157 | R01C01 |
| GSE117439 | GSM3294787 | sample 15, primary tumor from ERneg BC | case | 9422491156 | R01C01 |
| GSE117439 | GSM3294788 | sample 15, second tumor from ERneg BC | case | 9422491156 | R02C01 |
| GSE117439 | GSM3294789 | sample 16, primary tumor from ERneg BC | case | 9422491156 | R05C01 |
| GSE117439 | GSM3294790 | sample 16, second tumor from ERneg BC | case | 9422491156 | R04C02 |
| GSE117439 | GSM3294791 | sample 17, primary tumor from ERneg BC | case | 9421912123 | R06C02 |
| GSE117439 | GSM3294792 | sample 17, second tumor from ERneg BC | case | 9422492063 | R05C02 |
| GSE117439 | GSM3294793 | sample 18, primary tumor from ERpos BC | case | 9422491157 | R04C02 |
| GSE117439 | GSM3294794 | sample 18, second tumor from ERneg BC | case | 9422491157 | R03C02 |
| GSE117439 | GSM3294795 | sample 19, primary tumor from ERpos BC | case | 9422491157 | R05C02 |
| GSE117439 | GSM3294796 | sample 19, second tumor from ERneg BC | case | 9422492063 | R04C01 |
| GSE117439 | GSM3294797 | sample 20, primary tumor from ERpos BC | case | 9422492063 | R01C01 |
| GSE117439 | GSM3294798 | sample 20, second tumor from ERneg BC | case | 9422492137 | R04C01 |
| GSE117439 | GSM3294799 | sample 21, primary tumor from ERpos BC | case | 9422491157 | R03C01 |
| GSE117439 | GSM3294800 | sample 21, second tumor from ERneg BC | case | 9422492137 | R06C01 |
| GSE117439 | GSM3294801 | sample 22, primary tumor from ERpos BC | case | 9422492137 | R05C01 |
| GSE117439 | GSM3294802 | sample 22, second tumor from ERneg BC | case | 9422492063 | R06C02 |
| GSE117439 | GSM3294803 | sample 23, primary tumor from ERpos BC | case | 9422491157 | R02C02 |
| GSE117439 | GSM3294804 | sample 23, second tumor from ERneg BC | case | 9422491157 | R06C02 |
| GSE117439 | GSM3294805 | sample 24, primary tumor from ERpos BC | case | 9421912123 | R01C01 |
| GSE117439 | GSM3294806 | sample 25, primary tumor from ERpos BC | case | 9421912123 | R02C01 |
| GSE117439 | GSM3294807 | sample 26, primary tumor from ERpos BC | case | 9421912123 | R03C01 |
| GSE117439 | GSM3294808 | sample 27, primary tumor from ERpos BC | case | 9421912123 | R04C01 |
| GSE117439 | GSM3294809 | sample 28, primary tumor from ERpos BC | case | 9421912123 | R01C02 |
| GSE117439 | GSM3294810 | sample 29, primary tumor from ERpos BC | case | 9421912123 | R02C02 |
| GSE101961 | GSM2719518 | genomic DNA from breast tissue 6 | control | 101032610054 | R01C01 |
| GSE101961 | GSM2719519 | genomic DNA from breast tissue 21 | control | 101032610054 | R01C02 |
| GSE101961 | GSM2719520 | genomic DNA from breast tissue 9 | control | 101032610054 | R02C01 |
| GSE101961 | GSM2719521 | genomic DNA from breast tissue 22 | control | 101032610054 | R02C02 |
| GSE101961 | GSM2719522 | genomic DNA from breast tissue 14 | control | 101032610054 | R04C01 |
| GSE101961 | GSM2719523 | genomic DNA from breast tissue 36 | control | 101032610054 | R04C02 |
| GSE101961 | GSM2719524 | genomic DNA from breast tissue 19 | control | 101032610054 | R05C01 |
| GSE101961 | GSM2719525 | genomic DNA from breast tissue 41 | control | 101032610054 | R05C02 |
| GSE101961 | GSM2719526 | genomic DNA from breast tissue 20 | control | 101032610054 | R06C01 |
| GSE101961 | GSM2719527 | genomic DNA from breast tissue 70 | control | 101032610118 | R01C01 |
| GSE101961 | GSM2719528 | genomic DNA from breast tissue 104 | control | 101032610118 | R01C02 |
| GSE101961 | GSM2719529 | genomic DNA from breast tissue 75 | control | 101032610118 | R02C01 |
| GSE101961 | GSM2719530 | genomic DNA from breast tissue 106 | control | 101032610118 | R02C02 |
| GSE101961 | GSM2719531 | genomic DNA from breast tissue 76 | control | 101032610118 | R03C01 |
| GSE101961 | GSM2719532 | genomic DNA from breast tissue 108 | control | 101032610118 | R03C02 |
| GSE101961 | GSM2719533 | genomic DNA from breast tissue 77 | control | 101032610118 | R04C01 |
| GSE101961 | GSM2719534 | genomic DNA from breast tissue 110 | control | 101032610118 | R04C02 |
| GSE101961 | GSM2719535 | genomic DNA from breast tissue 93 | control | 101032610118 | R05C01 |
| GSE101961 | GSM2719536 | genomic DNA from breast tissue 112 | control | 101032610118 | R05C02 |
| GSE101961 | GSM2719537 | genomic DNA from breast tissue 95 | control | 101032610118 | R06C01 |
| GSE101961 | GSM2719538 | genomic DNA from breast tissue 114 | control | 101032610118 | R06C02 |
| GSE101961 | GSM2719539 | genomic DNA from breast tissue 116 | control | 101274800034 | R01C01 |
| GSE101961 | GSM2719540 | genomic DNA from breast tissue 81 | control | 101274800034 | R01C02 |
| GSE101961 | GSM2719541 | genomic DNA from breast tissue 83 | control | 101274800034 | R02C02 |
| GSE101961 | GSM2719542 | genomic DNA from breast tissue 39 | control | 101274800034 | R03C01 |
| GSE101961 | GSM2719543 | genomic DNA from breast tissue 84 | control | 101274800034 | R03C02 |
| GSE101961 | GSM2719544 | genomic DNA from breast tissue 78 | control | 101274800034 | R04C01 |
| GSE101961 | GSM2719545 | genomic DNA from breast tissue 86 | control | 101274800034 | R04C02 |
| GSE101961 | GSM2719546 | genomic DNA from breast tissue 79 | control | 101274800034 | R05C01 |
| GSE101961 | GSM2719547 | genomic DNA from breast tissue 87 | control | 101274800034 | R05C02 |
| GSE101961 | GSM2719548 | genomic DNA from breast tissue 80 | control | 101274800034 | R06C01 |
| GSE101961 | GSM2719549 | genomic DNA from breast tissue 88 | control | 101274800034 | R06C02 |
| GSE101961 | GSM2719550 | genomic DNA from breast tissue 101 | control | 101274800036 | R01C01 |
| GSE101961 | GSM2719551 | genomic DNA from breast tissue 111 | control | 101274800036 | R01C02 |
| GSE101961 | GSM2719552 | genomic DNA from breast tissue 117 | control | 101274800036 | R02C01 |
| GSE101961 | GSM2719553 | genomic DNA from breast tissue 113 | control | 101274800036 | R02C02 |
| GSE101961 | GSM2719554 | genomic DNA from breast tissue 2 | control | 101274800036 | R03C02 |
| GSE101961 | GSM2719555 | genomic DNA from breast tissue 1 | control | 101274800036 | R04C01 |
| GSE101961 | GSM2719556 | genomic DNA from breast tissue 3 | control | 101274800036 | R04C02 |
| GSE101961 | GSM2719557 | genomic DNA from breast tissue 18 | control | 101274800036 | R06C01 |
| GSE101961 | GSM2719558 | genomic DNA from breast tissue 44 | control | 6055432002 | R01C01 |
| GSE101961 | GSM2719559 | genomic DNA from breast tissue 29 | control | 6055432002 | R03C01 |
| GSE101961 | GSM2719560 | genomic DNA from breast tissue 16 | control | 6055432002 | R03C02 |
| GSE101961 | GSM2719561 | genomic DNA from breast tissue 94 | control | 6055432002 | R06C01 |
| GSE101961 | GSM2719562 | genomic DNA from breast tissue 92 | control | 6055432002 | R06C02 |
| GSE101961 | GSM2719563 | genomic DNA from breast tissue 85 | control | 6055432004 | R03C02 |
| GSE101961 | GSM2719564 | genomic DNA from breast tissue 119 | control | 6055432004 | R04C02 |
| GSE101961 | GSM2719565 | genomic DNA from breast tissue 34 | control | 6055432004 | R05C02 |
| GSE101961 | GSM2719566 | genomic DNA from breast tissue 73 | control | 6055432007 | R01C02 |
| GSE101961 | GSM2719567 | genomic DNA from breast tissue 120 | control | 6055432007 | R02C02 |
| GSE101961 | GSM2719568 | genomic DNA from breast tissue 26 | control | 6055432007 | R06C02 |
| GSE101961 | GSM2719569 | genomic DNA from breast tissue 47 | control | 6055432010 | R02C02 |
| GSE101961 | GSM2719570 | genomic DNA from breast tissue 96 | control | 6055432010 | R04C02 |
| GSE101961 | GSM2719571 | genomic DNA from breast tissue 72 | control | 6055432010 | R05C02 |
| GSE101961 | GSM2719572 | genomic DNA from breast tissue 62 | control | 6055432013 | R02C01 |
| GSE101961 | GSM2719573 | genomic DNA from breast tissue 68 | control | 6055432013 | R02C02 |
| GSE101961 | GSM2719574 | genomic DNA from breast tissue 53 | control | 6055432016 | R01C02 |
| GSE101961 | GSM2719575 | genomic DNA from breast tissue 40 | control | 6055432016 | R02C01 |
| GSE101961 | GSM2719576 | genomic DNA from breast tissue 118 | control | 6055432016 | R03C01 |
| GSE101961 | GSM2719577 | genomic DNA from breast tissue 27 | control | 6055432016 | R04C02 |
| GSE101961 | GSM2719578 | genomic DNA from breast tissue 25 | control | 6055432017 | R03C01 |
| GSE101961 | GSM2719579 | genomic DNA from breast tissue 67 | control | 6055432017 | R05C02 |
| GSE101961 | GSM2719580 | genomic DNA from breast tissue 33 | control | 6055432017 | R06C02 |
| GSE101961 | GSM2719581 | genomic DNA from breast tissue 69 | control | 6055432019 | R02C01 |
| GSE101961 | GSM2719582 | genomic DNA from breast tissue 100 | control | 6055432019 | R04C01 |
| GSE101961 | GSM2719583 | genomic DNA from breast tissue 56 | control | 6055432019 | R05C02 |
| GSE101961 | GSM2719584 | genomic DNA from breast tissue 54 | control | 6055432020 | R05C01 |
| GSE101961 | GSM2719585 | genomic DNA from breast tissue 91 | control | 6055432020 | R05C02 |
| GSE101961 | GSM2719586 | genomic DNA from breast tissue 8 | control | 6055432025 | R01C02 |
| GSE101961 | GSM2719587 | genomic DNA from breast tissue 61 | control | 6055432025 | R03C01 |
| GSE101961 | GSM2719588 | genomic DNA from breast tissue 66 | control | 6055432025 | R04C01 |
| GSE101961 | GSM2719589 | genomic DNA from breast tissue 12 | control | 6055432025 | R05C02 |
| GSE101961 | GSM2719590 | genomic DNA from breast tissue 89 | control | 6055432028 | R02C01 |
| GSE101961 | GSM2719591 | genomic DNA from breast tissue 49 | control | 6055432028 | R03C02 |
| GSE101961 | GSM2719592 | genomic DNA from breast tissue 17 | control | 6055432028 | R06C01 |
| GSE101961 | GSM2719593 | genomic DNA from breast tissue 4 | control | 6055432030 | R02C01 |
| GSE101961 | GSM2719594 | genomic DNA from breast tissue 42 | control | 6055432030 | R03C02 |
| GSE101961 | GSM2719595 | genomic DNA from breast tissue 46 | control | 6055432030 | R05C02 |
| GSE101961 | GSM2719596 | genomic DNA from breast tissue 50 | control | 6055432032 | R02C02 |
| GSE101961 | GSM2719597 | genomic DNA from breast tissue 31 | control | 6055432032 | R03C02 |
| GSE101961 | GSM2719598 | genomic DNA from breast tissue 103 | control | 6055432032 | R05C01 |
| GSE101961 | GSM2719599 | genomic DNA from breast tissue 65 | control | 6055432033 | R02C01 |
| GSE101961 | GSM2719600 | genomic DNA from breast tissue 115 | control | 6055432033 | R02C02 |
| GSE101961 | GSM2719601 | genomic DNA from breast tissue 7 | control | 6055432033 | R03C01 |
| GSE101961 | GSM2719602 | genomic DNA from breast tissue 98 | control | 6055432041 | R01C02 |
| GSE101961 | GSM2719603 | genomic DNA from breast tissue 60 | control | 6055432041 | R02C01 |
| GSE101961 | GSM2719604 | genomic DNA from breast tissue 52 | control | 6055432041 | R02C02 |
| GSE101961 | GSM2719605 | genomic DNA from breast tissue 57 | control | 6055432041 | R03C01 |
| GSE101961 | GSM2719606 | genomic DNA from breast tissue 43 | control | 6055432041 | R03C02 |
| GSE101961 | GSM2719607 | genomic DNA from breast tissue 97 | control | 6055432041 | R04C02 |
| GSE101961 | GSM2719608 | genomic DNA from breast tissue 45 | control | 6055432042 | R02C01 |
| GSE101961 | GSM2719609 | genomic DNA from breast tissue 32 | control | 6055432042 | R06C01 |
| GSE101961 | GSM2719610 | genomic DNA from breast tissue 38 | control | 6055432045 | R04C01 |
| GSE101961 | GSM2719611 | genomic DNA from breast tissue 105 | control | 6055432045 | R05C02 |
| GSE101961 | GSM2719612 | genomic DNA from breast tissue 23 | control | 6055432045 | R06C01 |
| GSE101961 | GSM2719613 | genomic DNA from breast tissue 99 | control | 6055432052 | R01C02 |
| GSE101961 | GSM2719614 | genomic DNA from breast tissue 28 | control | 6055432052 | R02C01 |
| GSE101961 | GSM2719615 | genomic DNA from breast tissue 48 | control | 6055432052 | R06C02 |
| GSE101961 | GSM2719616 | genomic DNA from breast tissue 63 | control | 6055432053 | R02C01 |
| GSE101961 | GSM2719617 | genomic DNA from breast tissue 24 | control | 6055432053 | R02C02 |
| GSE101961 | GSM2719618 | genomic DNA from breast tissue 11 | control | 6055432053 | R04C02 |
| GSE101961 | GSM2719619 | genomic DNA from breast tissue 35 | control | 6055432053 | R06C01 |
| GSE101961 | GSM2719620 | genomic DNA from breast tissue 5 | control | 6055432059 | R01C02 |
| GSE101961 | GSM2719621 | genomic DNA from breast tissue 64 | control | 6055432059 | R03C02 |
| GSE101961 | GSM2719622 | genomic DNA from breast tissue 90 | control | 6055432059 | R05C01 |
| GSE101961 | GSM2719623 | genomic DNA from breast tissue 58 | control | 6055432059 | R06C01 |
| GSE101961 | GSM2719624 | genomic DNA from breast tissue 51 | control | 6055432061 | R02C01 |
| GSE101961 | GSM2719625 | genomic DNA from breast tissue 121 | control | 6055432061 | R02C02 |
| GSE101961 | GSM2719626 | genomic DNA from breast tissue 55 | control | 6055432061 | R03C01 |
| GSE101961 | GSM2719627 | genomic DNA from breast tissue 37 | control | 6055432061 | R04C01 |
| GSE101961 | GSM2719628 | genomic DNA from breast tissue 30 | control | 6055432064 | R02C02 |
| GSE101961 | GSM2719629 | genomic DNA from breast tissue 74 | control | 6055432064 | R03C01 |
| GSE101961 | GSM2719630 | genomic DNA from breast tissue 15 | control | 6055432064 | R03C02 |
| GSE101961 | GSM2719631 | genomic DNA from breast tissue 71 | control | 6055432064 | R06C01 |
| GSE101961 | GSM2719632 | genomic DNA from breast tissue 109 | control | 6055432064 | R06C02 |
| GSE101961 | GSM2719633 | genomic DNA from breast tissue 10 | control | 6055432070 | R04C01 |
| GSE101961 | GSM2719634 | genomic DNA from breast tissue 13 | control | 6055432070 | R05C02 |
| GSE101961 | GSM2719635 | genomic DNA from breast tissue 102 | control | 6055432073 | R05C01 |
| GSE101961 | GSM2719636 | genomic DNA from breast tissue 82 | control | 6055432073 | R05C02 |
| GSE101961 | GSM2719637 | genomic DNA from breast tissue 59 | control | 6055432073 | R06C01 |
| GSE101961 | GSM2719638 | genomic DNA from breast tissue 107 | control | 6055432073 | R06C02 |
| GSE41169 | GSM1009660 | genomic DNA from whole blood 6 | whole blood |  |  |
| GSE41169 | GSM1009661 | genomic DNA from whole blood 7 | whole blood |  |  |
| GSE41169 | GSM1009662 | genomic DNA from whole blood 8 | whole blood |  |  |
| GSE41169 | GSM1009663 | genomic DNA from whole blood 9 | whole blood |  |  |
| GSE41169 | GSM1009664 | genomic DNA from whole blood 10 | whole blood |  |  |
| GSE41169 | GSM1009665 | genomic DNA from whole blood 11 | whole blood |  |  |
| GSE41169 | GSM1009666 | genomic DNA from whole blood 12 | whole blood |  |  |
| GSE41169 | GSM1009667 | genomic DNA from whole blood 13 | whole blood |  |  |
| GSE41169 | GSM1009668 | genomic DNA from whole blood 14 | whole blood |  |  |
| GSE41169 | GSM1009669 | genomic DNA from whole blood 15 | whole blood |  |  |
| GSE41169 | GSM1009670 | genomic DNA from whole blood 16 | whole blood |  |  |
| GSE41169 | GSM1009671 | genomic DNA from whole blood 17 | whole blood |  |  |
| GSE41169 | GSM1009672 | genomic DNA from whole blood 18 | whole blood |  |  |
| GSE41169 | GSM1009673 | genomic DNA from whole blood 19 | whole blood |  |  |
| GSE41169 | GSM1009674 | genomic DNA from whole blood 20 | whole blood |  |  |
| GSE41169 | GSM1009675 | genomic DNA from whole blood 21 | whole blood |  |  |
| GSE41169 | GSM1009676 | genomic DNA from whole blood 22 | whole blood |  |  |
| GSE41169 | GSM1009677 | genomic DNA from whole blood 23 | whole blood |  |  |
| GSE41169 | GSM1009678 | genomic DNA from whole blood 24 | whole blood |  |  |
| GSE41169 | GSM1009679 | genomic DNA from whole blood 25 | whole blood |  |  |
| GSE41169 | GSM1009680 | genomic DNA from whole blood 26 | whole blood |  |  |
| GSE41169 | GSM1009681 | genomic DNA from whole blood 27 | whole blood |  |  |
| GSE41169 | GSM1009682 | genomic DNA from whole blood 28 | whole blood |  |  |
| GSE41169 | GSM1009683 | genomic DNA from whole blood 29 | whole blood |  |  |
| GSE41169 | GSM1009684 | genomic DNA from whole blood 30 | whole blood |  |  |
| GSE41169 | GSM1009685 | genomic DNA from whole blood 31 | whole blood |  |  |
| GSE41169 | GSM1009686 | genomic DNA from whole blood 32 | whole blood |  |  |
| GSE41169 | GSM1009687 | genomic DNA from whole blood 33 | whole blood |  |  |
| GSE41169 | GSM1009688 | genomic DNA from whole blood 34 | whole blood |  |  |
| GSE41169 | GSM1009689 | genomic DNA from whole blood 35 | whole blood |  |  |
| GSE41169 | GSM1009690 | genomic DNA from whole blood 36 | whole blood |  |  |
| GSE41169 | GSM1009691 | genomic DNA from whole blood 37 | whole blood |  |  |
| GSE41169 | GSM1009692 | genomic DNA from whole blood 38 | whole blood |  |  |
| GSE41169 | GSM1009693 | genomic DNA from whole blood 39 | whole blood |  |  |
| GSE41169 | GSM1009694 | genomic DNA from whole blood 40 | whole blood |  |  |
| GSE41169 | GSM1009695 | genomic DNA from whole blood 41 | whole blood |  |  |
| GSE41169 | GSM1009696 | genomic DNA from whole blood 42 | whole blood |  |  |
| GSE41169 | GSM1009697 | genomic DNA from whole blood 43 | whole blood |  |  |
| GSE41169 | GSM1009698 | genomic DNA from whole blood 44 | whole blood |  |  |
| GSE41169 | GSM1009699 | genomic DNA from whole blood 45 | whole blood |  |  |
| GSE41169 | GSM1009700 | genomic DNA from whole blood 46 | whole blood |  |  |
| GSE41169 | GSM1009701 | genomic DNA from whole blood 47 | whole blood |  |  |
| GSE41169 | GSM1009702 | genomic DNA from whole blood 48 | whole blood |  |  |
| GSE41169 | GSM1009703 | genomic DNA from whole blood 49 | whole blood |  |  |
| GSE41169 | GSM1009704 | genomic DNA from whole blood 50 | whole blood |  |  |
| GSE41169 | GSM1009705 | genomic DNA from whole blood 51 | whole blood |  |  |
| GSE41169 | GSM1009706 | genomic DNA from whole blood 52 | whole blood |  |  |
| GSE41169 | GSM1009707 | genomic DNA from whole blood 53 | whole blood |  |  |
| GSE41169 | GSM1009708 | genomic DNA from whole blood 54 | whole blood |  |  |
| GSE41169 | GSM1009709 | genomic DNA from whole blood 55 | whole blood |  |  |
| GSE41169 | GSM1009710 | genomic DNA from whole blood 56 | whole blood |  |  |
| GSE41169 | GSM1009711 | genomic DNA from whole blood 57 | whole blood |  |  |
| GSE41169 | GSM1009712 | genomic DNA from whole blood 58 | whole blood |  |  |
| GSE41169 | GSM1009713 | genomic DNA from whole blood 59 | whole blood |  |  |
| GSE41169 | GSM1009714 | genomic DNA from whole blood 60 | whole blood |  |  |
| GSE41169 | GSM1009715 | genomic DNA from whole blood 61 | whole blood |  |  |
| GSE41169 | GSM1009716 | genomic DNA from whole blood 62 | whole blood |  |  |
| GSE41169 | GSM1009717 | genomic DNA from whole blood 63 | whole blood |  |  |
| GSE41169 | GSM1009718 | genomic DNA from whole blood 64 | whole blood |  |  |
| GSE41169 | GSM1009719 | genomic DNA from whole blood 65 | whole blood |  |  |
| GSE41169 | GSM1009720 | genomic DNA from whole blood 66 | whole blood |  |  |
| GSE41169 | GSM1009721 | genomic DNA from whole blood 67 | whole blood |  |  |
| GSE41169 | GSM1009722 | genomic DNA from whole blood 68 | whole blood |  |  |
| GSE41169 | GSM1009723 | genomic DNA from whole blood 69 | whole blood |  |  |
| GSE41169 | GSM1009724 | genomic DNA from whole blood 70 | whole blood |  |  |
| GSE41169 | GSM1009725 | genomic DNA from whole blood 71 | whole blood |  |  |
| GSE41169 | GSM1009726 | genomic DNA from whole blood 72 | whole blood |  |  |
| GSE41169 | GSM1009727 | genomic DNA from whole blood 73 | whole blood |  |  |
| GSE41169 | GSM1009728 | genomic DNA from whole blood 74 | whole blood |  |  |
| GSE41169 | GSM1009729 | genomic DNA from whole blood 75 | whole blood |  |  |
| GSE41169 | GSM1009730 | genomic DNA from whole blood 76 | whole blood |  |  |
| GSE41169 | GSM1009731 | genomic DNA from whole blood 77 | whole blood |  |  |
| GSE41169 | GSM1009732 | genomic DNA from whole blood 78 | whole blood |  |  |
| GSE41169 | GSM1009733 | genomic DNA from whole blood 79 | whole blood |  |  |
| GSE41169 | GSM1009734 | genomic DNA from whole blood 80 | whole blood |  |  |
| GSE41169 | GSM1009735 | genomic DNA from whole blood 81 | whole blood |  |  |
| GSE41169 | GSM1009736 | genomic DNA from whole blood 82 | whole blood |  |  |
| GSE41169 | GSM1009737 | genomic DNA from whole blood 83 | whole blood |  |  |
| GSE41169 | GSM1009738 | genomic DNA from whole blood 84 | whole blood |  |  |
| GSE41169 | GSM1009739 | genomic DNA from whole blood 85 | whole blood |  |  |
| GSE41169 | GSM1009740 | genomic DNA from whole blood 86 | whole blood |  |  |
| GSE41169 | GSM1009741 | genomic DNA from whole blood 87 | whole blood |  |  |
| GSE41169 | GSM1009742 | genomic DNA from whole blood 88 | whole blood |  |  |
| GSE41169 | GSM1009743 | genomic DNA from whole blood 89 | whole blood |  |  |
| GSE41169 | GSM1009744 | genomic DNA from whole blood 90 | whole blood |  |  |
| GSE41169 | GSM1009745 | genomic DNA from whole blood 91 | whole blood |  |  |
| GSE41169 | GSM1009746 | genomic DNA from whole blood 92 | whole blood |  |  |
| GSE41169 | GSM1009747 | genomic DNA from whole blood 93 | whole blood |  |  |
| GSE41169 | GSM1009748 | genomic DNA from whole blood 94 | whole blood |  |  |
| GSE41169 | GSM1009749 | genomic DNA from whole blood 95 | whole blood |  |  |
| GSE41169 | GSM1009890 | genomic DNA from whole blood 1 | whole blood |  |  |
| GSE41169 | GSM1009891 | genomic DNA from whole blood 2 | whole blood |  |  |
| GSE41169 | GSM1009892 | genomic DNA from whole blood 3 | whole blood |  |  |
| GSE41169 | GSM1009893 | genomic DNA from whole blood 4 | whole blood |  |  |
| GSE41169 | GSM1009894 | genomic DNA from whole blood 5 | whole blood |  |  |

Table S2 a total of 23 differentially methylated sites were identified.

| Gene | group | ADCY4 | CPXM1 | DNM3 | GNG4 | MAST1 | MIR129-2 | PRDM14 | PRKCB | ZNF177 |
| --- | --- | --- | --- | --- | --- | --- | --- | --- | --- | --- |
| GSM1858429 | case | 0.5000 | 0.5163 | 0.4923 | 0.4752 | 0.4706 | 0.3790 | 0.4863 | 0.3209 | 0.5895 |
| GSM1858430 | case | 0.4961 | 0.4652 | 0.4101 | 0.4057 | 0.4471 | 0.3108 | 0.4975 | 0.1339 | 0.4879 |
| GSM1858431 | case | 0.6968 | 0.6425 | 0.6616 | 0.6703 | 0.6251 | 0.6026 | 0.0841 | 0.4048 | 0.4722 |
| GSM1858432 | case | 0.8011 | 0.7808 | 0.8250 | 0.7887 | 0.7453 | 0.7583 | 0.7066 | 0.6956 | 0.7092 |
| GSM1858433 | case | 0.1289 | 0.0583 | 0.1618 | 0.3496 | 0.0504 | 0.0442 | 0.0591 | 0.0563 | 0.0861 |
| GSM1858434 | case | 0.1049 | 0.3963 | 0.2934 | 0.4589 | 0.4484 | 0.2815 | 0.5585 | 0.1866 | 0.5203 |
| GSM1858435 | case | 0.5362 | 0.3638 | 0.3164 | 0.3110 | 0.4589 | 0.2047 | 0.4948 | 0.2964 | 0.3635 |
| GSM1858436 | case | 0.4336 | 0.4676 | 0.5728 | 0.4619 | 0.4475 | 0.4513 | 0.5345 | 0.2667 | 0.6282 |
| GSM1858437 | case | 0.5327 | 0.5636 | 0.6622 | 0.6377 | 0.5969 | 0.5455 | 0.4823 | 0.4526 | 0.6711 |
| GSM1858438 | case | 0.0882 | 0.6012 | 0.0504 | 0.7122 | 0.6873 | 0.0603 | 0.0827 | 0.0340 | 0.0678 |
| GSM1858439 | case | 0.2651 | 0.5681 | 0.5750 | 0.4605 | 0.3603 | 0.5038 | 0.5545 | 0.0705 | 0.5414 |
| GSM1858440 | case | 0.5341 | 0.5841 | 0.5811 | 0.5820 | 0.5144 | 0.4752 | 0.4992 | 0.3432 | 0.6157 |
| GSM1858441 | case | 0.5144 | 0.5015 | 0.5561 | 0.5663 | 0.3888 | 0.4064 | 0.4934 | 0.3353 | 0.5697 |
| GSM1858442 | case | 0.5970 | 0.3110 | 0.6069 | 0.5544 | 0.3785 | 0.4514 | 0.3618 | 0.2954 | 0.5197 |
| GSM1858443 | case | 0.5887 | 0.6000 | 0.6785 | 0.6175 | 0.5486 | 0.4872 | 0.6594 | 0.3788 | 0.6030 |
| GSM1858444 | case | 0.5945 | 0.4038 | 0.7249 | 0.6542 | 0.6557 | 0.4716 | 0.3661 | 0.3742 | 0.1504 |
| GSM1858445 | case | 0.0776 | 0.4502 | 0.0545 | 0.1422 | 0.0599 | 0.3247 | 0.1624 | 0.1761 | 0.2651 |
| GSM1858446 | case | 0.1942 | 0.4851 | 0.6412 | 0.4862 | 0.4101 | 0.5383 | 0.3511 | 0.4701 | 0.6328 |
| GSM1858447 | case | 0.7125 | 0.3819 | 0.7438 | 0.4410 | 0.5563 | 0.4994 | 0.3916 | 0.2978 | 0.5157 |
| GSM1858448 | case | 0.7784 | 0.7214 | 0.8112 | 0.7763 | 0.7160 | 0.6660 | 0.7169 | 0.6086 | 0.7835 |
| GSM1858449 | case | 0.6623 | 0.4372 | 0.6968 | 0.4226 | 0.3449 | 0.3760 | 0.3428 | 0.5639 | 0.6548 |
| GSM1858450 | case | 0.7011 | 0.6292 | 0.5512 | 0.6631 | 0.6100 | 0.5343 | 0.5661 | 0.4807 | 0.6697 |
| GSM1858451 | case | 0.0864 | 0.1203 | 0.0725 | 0.1733 | 0.4487 | 0.1829 | 0.1659 | 0.1236 | 0.3605 |
| GSM1858452 | case | 0.4134 | 0.5250 | 0.5330 | 0.5216 | 0.5467 | 0.5008 | 0.5409 | 0.3587 | 0.6136 |
| GSM1858453 | case | 0.2832 | 0.5411 | 0.5441 | 0.4010 | 0.1025 | 0.3684 | 0.3626 | 0.3848 | 0.4470 |
| GSM1858454 | case | 0.0834 | 0.0799 | 0.0706 | 0.4200 | 0.4978 | 0.4462 | 0.1682 | 0.2888 | 0.6206 |
| GSM1858455 | case | 0.0745 | 0.0537 | 0.0588 | 0.1216 | 0.1094 | 0.0629 | 0.1067 | 0.0414 | 0.1827 |
| GSM1858456 | case | 0.4514 | 0.6297 | 0.7652 | 0.1982 | 0.1325 | 0.1879 | 0.3275 | 0.5313 | 0.4978 |
| GSM1858457 | case | 0.4912 | 0.5274 | 0.5782 | 0.5729 | 0.4902 | 0.4290 | 0.3095 | 0.2763 | 0.4770 |
| GSM1858458 | case | 0.6025 | 0.2217 | 0.6115 | 0.5708 | 0.4668 | 0.5687 | 0.5588 | 0.4578 | 0.6753 |
| GSM1858459 | case | 0.8507 | 0.7410 | 0.8714 | 0.8077 | 0.7349 | 0.7110 | 0.6388 | 0.5287 | 0.6279 |
| GSM1858460 | case | 0.5206 | 0.5817 | 0.7401 | 0.7380 | 0.5092 | 0.5528 | 0.6309 | 0.3196 | 0.7337 |
| GSM1858461 | case | 0.0730 | 0.1507 | 0.1180 | 0.6241 | 0.5211 | 0.0669 | 0.1562 | 0.0462 | 0.1684 |
| GSM1858462 | case | 0.0766 | 0.0795 | 0.0654 | 0.2502 | 0.3323 | 0.2081 | 0.2308 | 0.1743 | 0.1321 |
| GSM1858463 | case | 0.7816 | 0.7151 | 0.7707 | 0.7883 | 0.6874 | 0.7094 | 0.7264 | 0.4990 | 0.2017 |
| GSM1858464 | case | 0.3198 | 0.2677 | 0.3161 | 0.4365 | 0.2505 | 0.4193 | 0.4417 | 0.0821 | 0.6084 |
| GSM1858465 | case | 0.4017 | 0.2805 | 0.4112 | 0.5247 | 0.2697 | 0.4751 | 0.4501 | 0.0860 | 0.6370 |
| GSM1858466 | case | 0.5825 | 0.5298 | 0.2574 | 0.5438 | 0.4861 | 0.4781 | 0.5546 | 0.3549 | 0.2242 |
| GSM1858467 | case | 0.2200 | 0.4810 | 0.0847 | 0.4746 | 0.3009 | 0.1614 | 0.2364 | 0.1139 | 0.2087 |
| GSM1858468 | case | 0.0953 | 0.4105 | 0.0953 | 0.1693 | 0.2500 | 0.4163 | 0.5014 | 0.2988 | 0.5706 |
| GSM1858469 | case | 0.5777 | 0.5778 | 0.6355 | 0.5209 | 0.5231 | 0.5029 | 0.5997 | 0.4088 | 0.4534 |
| GSM1858470 | case | 0.0851 | 0.0767 | 0.0725 | 0.1081 | 0.1886 | 0.6624 | 0.4459 | 0.1507 | 0.1231 |
| GSM1858471 | case | 0.2677 | 0.3080 | 0.1391 | 0.0878 | 0.2183 | 0.4219 | 0.2530 | 0.1912 | 0.5203 |
| GSM1858472 | case | 0.0832 | 0.0720 | 0.0799 | 0.0980 | 0.5314 | 0.0864 | 0.6019 | 0.0588 | 0.1692 |
| GSM1858473 | case | 0.6872 | 0.6111 | 0.6912 | 0.6972 | 0.5811 | 0.5888 | 0.5806 | 0.6985 | 0.6232 |
| GSM1858474 | case | 0.7029 | 0.6406 | 0.7023 | 0.7184 | 0.6019 | 0.6357 | 0.6443 | 0.5291 | 0.5519 |
| GSM1858475 | case | 0.7204 | 0.6981 | 0.6979 | 0.7333 | 0.6034 | 0.6091 | 0.6818 | 0.5017 | 0.7452 |
| GSM1858476 | case | 0.0595 | 0.0520 | 0.0632 | 0.0770 | 0.7386 | 0.0540 | 0.0771 | 0.0327 | 0.0777 |
| GSM1858477 | case | 0.6225 | 0.5945 | 0.6598 | 0.5525 | 0.5361 | 0.2491 | 0.6613 | 0.3581 | 0.6148 |
| GSM1858478 | case | 0.6120 | 0.5767 | 0.5185 | 0.6073 | 0.5757 | 0.5111 | 0.6290 | 0.4137 | 0.6590 |
| GSM1858479 | case | 0.5908 | 0.6777 | 0.6570 | 0.6068 | 0.0825 | 0.4818 | 0.6418 | 0.5795 | 0.5390 |
| GSM1858480 | case | 0.7223 | 0.3613 | 0.6766 | 0.7622 | 0.5735 | 0.6140 | 0.4207 | 0.6425 | 0.7208 |
| GSM1858481 | case | 0.1083 | 0.2640 | 0.0972 | 0.1670 | 0.4151 | 0.0975 | 0.1788 | 0.0545 | 0.2718 |
| GSM1858482 | case | 0.0781 | 0.0638 | 0.0668 | 0.0910 | 0.0844 | 0.0665 | 0.0675 | 0.3269 | 0.0847 |
| GSM1858483 | case | 0.1029 | 0.2819 | 0.3119 | 0.0973 | 0.2567 | 0.2846 | 0.2368 | 0.3645 | 0.3485 |
| GSM1858484 | case | 0.2185 | 0.2025 | 0.5827 | 0.2565 | 0.3246 | 0.6420 | 0.6073 | 0.6003 | 0.5892 |
| GSM1858485 | case | 0.5949 | 0.5003 | 0.6499 | 0.7044 | 0.3971 | 0.5526 | 0.5151 | 0.3980 | 0.5835 |
| GSM1858486 | case | 0.6783 | 0.5463 | 0.6348 | 0.5184 | 0.5602 | 0.1722 | 0.2349 | 0.3095 | 0.6773 |
| GSM1858487 | case | 0.1282 | 0.1227 | 0.0860 | 0.1119 | 0.0841 | 0.1347 | 0.2622 | 0.4415 | 0.7294 |
| GSM1858488 | case | 0.6360 | 0.1899 | 0.5828 | 0.4891 | 0.5149 | 0.2137 | 0.4245 | 0.4329 | 0.5759 |
| GSM1858489 | case | 0.6075 | 0.5221 | 0.3206 | 0.6871 | 0.5177 | 0.3960 | 0.5311 | 0.2553 | 0.1269 |
| GSM1858490 | case | 0.2993 | 0.1137 | 0.1864 | 0.3637 | 0.0811 | 0.2548 | 0.1882 | 0.0873 | 0.5033 |
| GSM1858491 | case | 0.6607 | 0.6589 | 0.4954 | 0.5548 | 0.4444 | 0.4951 | 0.1934 | 0.5759 | 0.6228 |
| GSM1858492 | case | 0.1100 | 0.0885 | 0.0995 | 0.1417 | 0.1357 | 0.1022 | 0.1584 | 0.1264 | 0.2529 |
| GSM1858493 | case | 0.5469 | 0.4860 | 0.5004 | 0.5383 | 0.5261 | 0.4961 | 0.5656 | 0.2793 | 0.5487 |
| GSM1858494 | case | 0.5663 | 0.5383 | 0.6078 | 0.5474 | 0.5256 | 0.5535 | 0.5968 | 0.3125 | 0.6007 |
| GSM1858495 | case | 0.7716 | 0.7476 | 0.8189 | 0.7954 | 0.6679 | 0.6094 | 0.6212 | 0.5527 | 0.7634 |
| GSM1858496 | case | 0.0857 | 0.0749 | 0.5450 | 0.1061 | 0.0787 | 0.4966 | 0.3623 | 0.6015 | 0.3893 |
| GSM1858497 | case | 0.6366 | 0.6240 | 0.6135 | 0.5108 | 0.2455 | 0.5817 | 0.6240 | 0.5028 | 0.7029 |
| GSM1858498 | case | 0.5373 | 0.5638 | 0.0658 | 0.6217 | 0.4807 | 0.5165 | 0.6343 | 0.4225 | 0.1646 |
| GSM1858499 | case | 0.4872 | 0.5332 | 0.6722 | 0.6063 | 0.5446 | 0.5251 | 0.5630 | 0.2631 | 0.6467 |
| GSM1858500 | case | 0.7667 | 0.7142 | 0.8096 | 0.7925 | 0.4964 | 0.5883 | 0.5725 | 0.7258 | 0.6657 |
| GSM1858501 | case | 0.5187 | 0.4939 | 0.6974 | 0.5909 | 0.3045 | 0.4014 | 0.1525 | 0.6135 | 0.6318 |
| GSM1858502 | case | 0.2790 | 0.4552 | 0.3425 | 0.2743 | 0.2555 | 0.1954 | 0.4242 | 0.0672 | 0.4116 |
| GSM1858503 | case | 0.3411 | 0.4138 | 0.3156 | 0.1907 | 0.2967 | 0.2867 | 0.3997 | 0.1819 | 0.3926 |
| GSM1858504 | case | 0.1172 | 0.4606 | 0.0815 | 0.4706 | 0.4126 | 0.2800 | 0.4514 | 0.1739 | 0.4781 |
| GSM1858505 | case | 0.5650 | 0.6144 | 0.6991 | 0.5290 | 0.5371 | 0.0860 | 0.4430 | 0.4989 | 0.5948 |
| GSM1858506 | case | 0.4853 | 0.5847 | 0.6539 | 0.6352 | 0.4391 | 0.4177 | 0.3695 | 0.4146 | 0.5713 |
| GSM1858507 | case | 0.6945 | 0.4025 | 0.7258 | 0.5951 | 0.4075 | 0.5646 | 0.3802 | 0.4247 | 0.5950 |
| GSM1858508 | case | 0.0900 | 0.5100 | 0.5095 | 0.5288 | 0.4780 | 0.4011 | 0.4726 | 0.3316 | 0.5378 |
| GSM1858509 | case | 0.6512 | 0.5943 | 0.7366 | 0.6370 | 0.4056 | 0.5767 | 0.6159 | 0.6263 | 0.6272 |
| GSM1858510 | case | 0.0891 | 0.0762 | 0.0789 | 0.0901 | 0.0758 | 0.0631 | 0.1238 | 0.0634 | 0.2232 |
| GSM1858511 | case | 0.5933 | 0.4681 | 0.6366 | 0.5135 | 0.3651 | 0.6480 | 0.6922 | 0.4982 | 0.7426 |
| GSM1858512 | case | 0.5853 | 0.5293 | 0.6018 | 0.4169 | 0.4877 | 0.5083 | 0.5747 | 0.3961 | 0.6061 |
| GSM1858513 | case | 0.0779 | 0.3897 | 0.5157 | 0.3970 | 0.1008 | 0.6053 | 0.6424 | 0.6181 | 0.6135 |
| GSM1858514 | case | 0.0979 | 0.3821 | 0.5627 | 0.6191 | 0.3932 | 0.4507 | 0.3606 | 0.6455 | 0.6259 |
| GSM1858515 | case | 0.3727 | 0.5944 | 0.4804 | 0.5995 | 0.4868 | 0.4714 | 0.4001 | 0.4529 | 0.6316 |
| GSM1858516 | case | 0.6028 | 0.6049 | 0.7031 | 0.6999 | 0.4100 | 0.3914 | 0.3025 | 0.8062 | 0.5499 |
| GSM1858517 | case | 0.7235 | 0.6669 | 0.7797 | 0.5323 | 0.5790 | 0.6738 | 0.5362 | 0.5982 | 0.5253 |
| GSM1858518 | case | 0.0985 | 0.1685 | 0.0832 | 0.1174 | 0.0718 | 0.2421 | 0.3359 | 0.0828 | 0.2046 |
| GSM1858519 | case | 0.2305 | 0.0840 | 0.5954 | 0.6593 | 0.2707 | 0.3988 | 0.1343 | 0.4391 | 0.4932 |
| GSM1858520 | case | 0.7130 | 0.4189 | 0.7381 | 0.5011 | 0.3324 | 0.6478 | 0.5556 | 0.6129 | 0.6403 |
| GSM1858521 | case | 0.6681 | 0.5358 | 0.6949 | 0.3944 | 0.3028 | 0.5273 | 0.5744 | 0.4052 | 0.6590 |
| GSM1858522 | case | 0.6498 | 0.2623 | 0.6276 | 0.6689 | 0.2027 | 0.5393 | 0.4427 | 0.4637 | 0.5611 |
| GSM1858523 | case | 0.0935 | 0.5007 | 0.0679 | 0.0887 | 0.5777 | 0.0996 | 0.1420 | 0.3648 | 0.2118 |
| GSM1858524 | case | 0.2012 | 0.7108 | 0.7556 | 0.5113 | 0.3914 | 0.5898 | 0.5821 | 0.5971 | 0.7133 |
| GSM1858525 | case | 0.5949 | 0.5627 | 0.6558 | 0.6474 | 0.5205 | 0.3383 | 0.4965 | 0.5842 | 0.6063 |
| GSM1858526 | case | 0.7132 | 0.3720 | 0.5972 | 0.3189 | 0.0865 | 0.5350 | 0.6718 | 0.3979 | 0.1118 |
| GSM1858527 | case | 0.5870 | 0.3876 | 0.4850 | 0.3959 | 0.4655 | 0.3748 | 0.4467 | 0.2814 | 0.6015 |
| GSM1858528 | case | 0.6621 | 0.6274 | 0.7341 | 0.7054 | 0.6032 | 0.5797 | 0.5013 | 0.5826 | 0.6470 |
| GSM1858529 | case | 0.6328 | 0.6154 | 0.6538 | 0.6390 | 0.5804 | 0.5639 | 0.6525 | 0.5165 | 0.6333 |
| GSM1858530 | case | 0.7014 | 0.4738 | 0.7004 | 0.6649 | 0.5003 | 0.6276 | 0.4905 | 0.5156 | 0.6693 |
| GSM1858531 | case | 0.7333 | 0.5560 | 0.7681 | 0.7413 | 0.4630 | 0.2662 | 0.4421 | 0.4197 | 0.6935 |
| GSM1858532 | case | 0.5787 | 0.6081 | 0.2233 | 0.5645 | 0.5492 | 0.5607 | 0.5677 | 0.4408 | 0.6203 |
| GSM1858533 | case | 0.5567 | 0.5858 | 0.4376 | 0.5814 | 0.5280 | 0.5322 | 0.5478 | 0.2433 | 0.6178 |
| GSM1858534 | case | 0.6560 | 0.6197 | 0.3282 | 0.5146 | 0.4338 | 0.5237 | 0.6326 | 0.3508 | 0.1816 |
| GSM1858535 | case | 0.5251 | 0.7024 | 0.8143 | 0.7664 | 0.7364 | 0.7086 | 0.7689 | 0.2883 | 0.7847 |
| GSM1858536 | case | 0.1134 | 0.0909 | 0.0950 | 0.1645 | 0.1081 | 0.1048 | 0.2310 | 0.0507 | 0.2496 |
| GSM1858537 | case | 0.0879 | 0.5215 | 0.6617 | 0.5747 | 0.6134 | 0.5976 | 0.6939 | 0.6290 | 0.6903 |
| GSM1858538 | case | 0.4489 | 0.5671 | 0.7092 | 0.6926 | 0.4966 | 0.5522 | 0.5992 | 0.5266 | 0.1817 |
| GSM1858539 | case | 0.5122 | 0.3441 | 0.6779 | 0.6337 | 0.4427 | 0.5389 | 0.6061 | 0.5707 | 0.6718 |
| GSM1858540 | case | 0.5755 | 0.5669 | 0.5481 | 0.6298 | 0.4286 | 0.3872 | 0.1221 | 0.5188 | 0.5683 |
| GSM1858541 | case | 0.8417 | 0.7998 | 0.8481 | 0.8311 | 0.5888 | 0.7969 | 0.6556 | 0.7700 | 0.7808 |
| GSM1858542 | case | 0.4478 | 0.3336 | 0.7249 | 0.3529 | 0.2366 | 0.4273 | 0.4603 | 0.4724 | 0.6349 |
| GSM1858543 | case | 0.1173 | 0.4455 | 0.0747 | 0.6027 | 0.4484 | 0.3761 | 0.2610 | 0.0506 | 0.2205 |
| GSM1858544 | case | 0.5863 | 0.4505 | 0.6633 | 0.4218 | 0.2626 | 0.5003 | 0.3236 | 0.5716 | 0.5685 |
| GSM1858545 | case | 0.5710 | 0.4870 | 0.6146 | 0.5824 | 0.2087 | 0.4061 | 0.3356 | 0.4454 | 0.5954 |
| GSM1858546 | case | 0.6124 | 0.5618 | 0.7770 | 0.5904 | 0.4427 | 0.6029 | 0.6080 | 0.5457 | 0.7000 |
| GSM2350679 | control | 0.0335 | 0.0194 | 0.0294 | 0.0271 | 0.0189 | 0.0218 | 0.0284 |  | 0.0376 |
| GSM2350680 | control | 0.0538 | 0.0404 | 0.0491 | 0.0781 | 0.0251 | 0.0339 | 0.0496 |  | 0.0765 |
| GSM2350681 | control | 0.0306 | 0.0175 | 0.0234 | 0.0282 | 0.0193 | 0.0226 | 0.0241 |  | 0.0262 |
| GSM2350682 | control | 0.0462 | 0.0265 | 0.0380 | 0.0512 | 0.0407 | 0.0314 | 0.0390 |  | 0.0672 |
| GSM2350683 | control | 0.0399 | 0.0222 | 0.0313 | 0.0472 | 0.0351 | 0.0240 | 0.0690 |  | 0.0381 |
| GSM2350684 | control | 0.0837 | 0.0529 | 0.0483 | 0.1359 | 0.0177 | 0.0571 | 0.1198 |  | 0.1246 |
| GSM2350685 | control | 0.0352 | 0.0257 | 0.0281 | 0.0270 | 0.0273 | 0.0212 | 0.0359 |  | 0.0304 |
| GSM2350686 | control | 0.0495 | 0.0793 | 0.0368 | 0.0805 | 0.0194 | 0.0309 | 0.0607 |  | 0.0929 |
| GSM2350687 | control | 0.0636 | 0.0229 | 0.0352 | 0.0429 | 0.0222 | 0.0186 | 0.0344 |  | 0.0577 |
| GSM2350688 | control | 0.0285 | 0.0151 | 0.0314 | 0.0253 | 0.0201 | 0.0179 | 0.0296 |  | 0.0249 |
| GSM2350689 | control | 0.0431 | 0.0581 | 0.0369 | 0.0619 | 0.0493 | 0.0463 | 0.0869 |  | 0.1224 |
| GSM2350690 | control | 0.0384 | 0.0354 | 0.0420 | 0.0699 | 0.0197 | 0.0335 | 0.0559 |  | 0.0826 |
| GSM2350691 | control | 0.0363 | 0.0187 | 0.0254 | 0.0311 | 0.0231 | 0.0194 | 0.0246 |  | 0.0280 |
| GSM2350692 | control | 0.0393 | 0.0205 | 0.0364 | 0.0313 | 0.0483 | 0.0230 | 0.0456 |  | 0.0382 |
| GSM2350693 | control | 0.0462 | 0.0194 | 0.0256 | 0.1114 | 0.0249 | 0.0307 | 0.0811 |  | 0.0808 |
| GSM2350694 | control | 0.0396 | 0.0196 | 0.0360 | 0.0335 | 0.0251 | 0.0257 | 0.0429 |  | 0.0504 |
| GSM2350695 | control | 0.1534 | 0.0412 | 0.0527 | 0.0708 | 0.0196 | 0.0307 | 0.0666 |  | 0.1386 |
| GSM2350696 | control | 0.0356 | 0.0156 | 0.0366 | 0.0491 | 0.0199 | 0.0238 | 0.0424 |  | 0.0588 |
| GSM2350697 | control | 0.0429 | 0.0248 | 0.0307 | 0.0541 | 0.0236 | 0.0225 | 0.0228 |  | 0.0498 |
| GSM2350698 | control | 0.0646 | 0.0518 | 0.0339 | 0.0682 | 0.0216 | 0.0421 | 0.0404 |  | 0.0882 |
| GSM2350699 | control | 0.0363 | 0.0201 | 0.0246 | 0.0336 | 0.0227 | 0.0190 | 0.0331 |  | 0.0358 |
| GSM2350700 | control | 0.0743 | 0.0606 | 0.0511 | 0.0411 | 0.0192 | 0.0897 | 0.0526 |  | 0.1105 |
| GSM2350701 | control | 0.0358 | 0.0212 | 0.0277 | 0.0387 | 0.0211 | 0.0214 | 0.0287 |  | 0.0346 |
| GSM2350702 | control | 0.1104 | 0.0448 | 0.1128 | 0.0896 | 0.0227 | 0.0351 | 0.1075 |  | 0.1791 |
| GSM2350703 | control | 0.0545 | 0.0210 | 0.0317 | 0.0581 | 0.0216 | 0.0276 | 0.0377 |  | 0.0590 |
| GSM2350704 | control | 0.0328 | 0.0416 | 0.0426 | 0.0410 | 0.0169 | 0.0270 | 0.0411 |  | 0.0691 |
| GSM2350705 | control | 0.0439 | 0.0335 | 0.0457 | 0.0443 | 0.0280 | 0.0414 | 0.0459 |  | 0.0612 |
| GSM2350706 | control | 0.0287 | 0.0222 | 0.0253 | 0.0314 | 0.0252 | 0.0213 | 0.0335 |  | 0.0412 |
| GSM2350707 | control | 0.0448 | 0.0238 | 0.0259 | 0.0519 | 0.0581 | 0.0326 | 0.0559 |  | 0.0931 |
| GSM2350708 | control | 0.0942 | 0.0216 | 0.0459 | 0.0532 | 0.0229 | 0.0242 | 0.0327 |  | 0.0522 |
| GSM2350709 | control | 0.0571 | 0.0268 | 0.0284 | 0.0406 | 0.0255 | 0.0253 | 0.0355 |  | 0.0456 |
| GSM2350710 | control | 0.0295 | 0.0240 | 0.0267 | 0.0421 | 0.0206 | 0.0249 | 0.0309 |  | 0.2072 |
| GSM2350711 | control | 0.0336 | 0.0274 | 0.0304 | 0.0517 | 0.0218 | 0.0312 | 0.0662 |  | 0.0642 |
| GSM2350712 | control | 0.0377 | 0.0266 | 0.0349 | 0.0406 | 0.0839 | 0.0276 | 0.0409 |  | 0.0770 |
| GSM2350713 | control | 0.0526 | 0.0308 | 0.0332 | 0.0677 | 0.0227 | 0.0320 | 0.0756 |  | 0.1120 |
| GSM2350714 | control | 0.0583 | 0.0539 | 0.0384 | 0.0588 | 0.0553 | 0.0469 | 0.0712 |  | 0.0847 |
| GSM2350715 | control | 0.0468 | 0.0317 | 0.0301 | 0.0576 | 0.0209 | 0.0313 | 0.0541 |  | 0.0723 |
| GSM2350716 | control | 0.0456 | 0.0400 | 0.0320 | 0.1108 | 0.0351 | 0.0461 | 0.0880 |  | 0.1557 |
| GSM2350717 | control | 0.0548 | 0.0500 | 0.0328 | 0.0852 | 0.0255 | 0.0347 | 0.0747 |  | 0.0887 |
| GSM2350718 | control | 0.0799 | 0.0275 | 0.0406 | 0.0623 | 0.0207 | 0.0236 | 0.0533 |  | 0.0992 |
| GSM2350719 | control | 0.0320 | 0.0184 | 0.0273 | 0.0377 | 0.0272 | 0.0213 | 0.0307 |  | 0.0479 |
| GSM2350720 | control | 0.0756 | 0.0340 | 0.0345 | 0.0254 | 0.1072 | 0.0286 | 0.0374 |  | 0.0537 |
| GSM2350721 | control | 0.0469 | 0.0418 | 0.0593 | 0.0518 | 0.0193 | 0.0252 | 0.0320 |  | 0.0276 |
| GSM2350722 | control | 0.0887 | 0.0354 | 0.0462 | 0.0581 | 0.0402 | 0.0225 | 0.0474 |  | 0.0994 |
| GSM2350723 | control | 0.0681 | 0.0407 | 0.0439 | 0.0823 | 0.0315 | 0.0274 | 0.0414 |  | 0.1030 |
| GSM2350724 | control | 0.1095 | 0.0253 | 0.0366 | 0.0499 | 0.0238 | 0.0245 | 0.1172 |  | 0.2322 |
| GSM2350725 | control | 0.0222 | 0.0192 | 0.0237 | 0.0366 | 0.0202 | 0.0175 | 0.0253 |  | 0.0263 |
| GSM2350726 | control | 0.0292 | 0.0194 | 0.0256 | 0.0309 | 0.0204 | 0.0196 | 0.0262 |  | 0.0386 |
| GSM2350727 | control | 0.0642 | 0.0832 | 0.0441 | 0.1230 | 0.0316 | 0.0409 | 0.1078 |  | 0.2088 |
| GSM2350728 | control | 0.0441 | 0.0270 | 0.0415 | 0.0434 | 0.0231 | 0.0250 | 0.0362 |  | 0.0914 |
